# Newly Synthesized Fluorinated Cinnamylpiperazines Possessing Low In Vitro MAO-B Binding

**DOI:** 10.3390/molecules25214941

**Published:** 2020-10-26

**Authors:** Ivana I. Jevtić, Thu Hang Lai, Jelena Z. Penjišević, Sladjana Dukić-Stefanović, Deana B. Andrić, Peter Brust, Sladjana V. Kostić-Rajačić, Rodrigo Teodoro

**Affiliations:** 1 ICTM-Department of Chemistry, University of Belgrade, Njegoševa 12, 11000 Belgrade, Serbia; ivana.jevtic@ihtm.bg.ac.rs (I.I.J.); jelena.penjisevic@ihtm.bg.ac.rs (J.Z.P.); sladjana.kostic@ihtm.bg.ac.rs (S.V.K.-R.); 2 Department of Neuroradiopharmaceuticals, Institute of Radiopharmaceutical Cancer Research, Helmholtz-Zentrum Dresden-Rossendorf, Research Site Leipzig, Permoserstraße 15, 04318 Leipzig, Germany; t.lai@hzdr.de (T.H.L.); s.dukic-stefanovic@hzdr.de (S.D.-S.); p.brust@hzdr.de (P.B.); 3 Department of Research and Development, ROTOP Pharmaka GmbH, 01328 Dresden, Germany; 4 Faculty of Chemistry, University of Belgrade, Studentski trg 12-16, 11000 Belgrade, Serbia; deanad@chem.bg.ac.rs

**Keywords:** MAO-B, positron emission tomography, piperazine, cinnamic acid

## Abstract

Herein, we report on the synthesis and pharmacological evaluation of ten novel fluorinated cinnamylpiperazines as potential monoamine oxidase B (MAO-B) ligands. The designed derivatives consist of either cinnamyl or 2-fluorocinnamyl moieties connected to 2-fluoropyridylpiperazines. The three-step synthesis starting from commercially available piperazine afforded the final products in overall yields between 9% and 29%. An in vitro competitive binding assay using l-[^3^H]Deprenyl as radioligand was developed and the MAO-B binding affinities of the synthesized derivatives were assessed. Docking studies revealed that the compounds **8**–**17** were stabilized in both MAO-B entrance and substrate cavities, thus resembling the binding pose of l-Deprenyl. Although our results revealed that the novel fluorinated cinnamylpiperazines **8**–**17** do not possess sufficient MAO-B binding affinity to be eligible as positron emission tomography (PET) agents, the herein developed binding assay and the insights gained within our docking studies will certainly pave the way for further development of MAO-B ligands.

## 1. Introduction

Monoamine oxidase (MAO) belongs to a family of flavin-containing enzymes that are present in the outer mitochondrial membrane in neurons and glial cells in the central nervous system, although a small proportion is associated with the microsomal fraction [1,2]. These enzymes have an important role in the oxidative deamination of a variety of biogenic and xenobiotic amines, thereby influencing their availability and physiological activity in the brain and peripheral tissues [3]. There are two isoforms identified so far, MAO-A and MAO-B, which are encoded by independent genes, possess different regional and cellular distributions, and exhibit distinct selectivity toward various monoamine neurotransmitters [4]. Although they share 70% of amino acid sequence identity, MAO-A selectively oxidizes serotonin, while β-phenethylamine and benzylamine are selective substrates for MAO-B [5].

In the human brain, ∼70% of total MAO activity is regarded to MAO-B, and, during the normal aging process, its availability increases at the rate of nearly 9% per decade [6,7]. In fact, MAO-B inhibitors have been proposed as biomarkers for astrocytosis in neurodegenerative diseases associated with cell death or the activation of immune responses, with some investigations reporting higher MAO-B binding in Alzheimer’s disease patients versus aged-matched controls [8,9]. Moreover, MAO-B inhibition has significant therapeutic effects for mood disorders and Parkinson’s disease [10]. Significantly higher activity levels of MAO-B have also been reported in glioblastoma, low-grade astrocytomas, and anaplastic astrocytomas compared to those of post-mortem control brain [11].

In sight of the involvement of the MAO-B enzyme in crucial pathophysiological processes, the development of inhibitors targeting MAO-B for therapeutic and diagnostic purposes has been the aim of several pharmaceutical companies as well as academic institutions [4]. Amongst all chemical classes already disclosed, indanone, coumarin, acetophenone and benzothiazole derivatives are examples of highly affine and selective scaffolds with potential to target MAO-B. For example, the FDA-approved drug Selegiline^®^, also known as l-Deprenyl, has been used in the adjuvant treatment of Parkinson’s disease [12].

**Figure 1:**
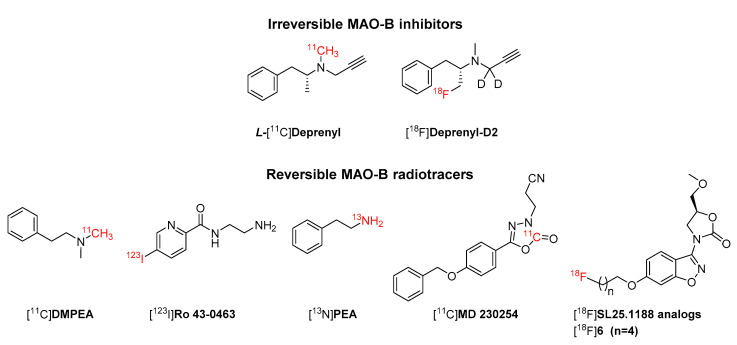
Representative irreversible and reversible monoamine oxidase B (MAO-B) imaging agents.

To track the efficacy of these MAO-B tailored therapies and also to monitor the changes in the receptor availability during disease progression, attempts to develop positron emission tomography (PET) imaging agents were also extensively explored (Figure 1) [13,14]. Fowler et al. [5] classified the MAO-B imaging agents according to their mode of action, namely the irreversible and the reversible binders. So far, the irreversible binders l-[^11^C]Deprenyl [8] and its isotopologue, l-[^11^C]Deprenyl-D2, are the only MAO-B radioligands evaluated in clinical trials [15,16]. However, as the short half-life of carbon-11 (^11^C, 20.4 min) makes these tracers less attractive for distribution to PET centers, the development of an ^18^F-labeled deuterated analog of l-Deprenyl was proposed as an alternative [16].

l-[^18^F]Deprenyl presented a moderate in vitro inhibitory potency against the recombinant human MAO-B (IC_50, MAO-B_ = 227 ± 36.8 nM). Although in vitro autoradiography in healthy human brain as well as in vivo experiments in a cynomolgus monkey revealed a selective binding to MAO-B-rich brain regions, such as striatum, the presence of blood–brain barrier (BBB) penetrant radiometabolites hindered its further evaluation. The deuterated l-[^18^F]Deprenyl-D2 was employed as an alternative to increase the in vivo stability of l-[^18^F]Deprenyl; however, the presence of [^18^F]fluorometamphetamine-D2 in brain was questionable [17]. Attempts to explore the reversible binders from different chemical classes labeled with different radionuclides (Figure 1) have not outperformed l-[^11^C]Deprenyl-D2. Most recently, the oxazolidine [^18^F]FSL25.1188 and its analogs were also investigated [18]. Among them, the analog possessing a fluoropentoxy alky chain, namely [^18^F]**6** (IC_50, MAO-B_ = 110 nM, Figure 1), presented a better pharmacokinetic profile and in vivo stability [18].

Although some of the above-mentioned radioligands might be putative candidates, their rather low potency towards MAO-B and poor selectivity towards MAO-A are the major limiting factors during imaging quantification of MAO-B enzymatic activity in brain. In particular, for PET ligands, attention has to be given to the ratio of the target receptor density (*B_max_*) to the target affinity of a given compound (*K_D_*). In general, a *B_max_/K_D_* ratio greater than 10 should provide a useful specific signal in vivo [19].

The combination of different promising pharmacophores is a very useful concept for the rational design of new biologically active compounds [20], as illustrated by the pyridoxine-resveratrol [21] and pyridazine-coumarin [22] hybrids developed for MAO-B. While arylpiperazine has been identified as a common structural motif in antipsychotic drugs used for the treatment of neurodegenerative disorders [23] and also as a benign prostatic hyperplasia or hypertension antagonists mediated by α1 adrenergic receptors [24], the brain penetrant cinnamyl acids bearing *N*-benzyl pyridinium derivatives were reported as multifunctional cholinesterase inhibitors against Alzheimer’s disease by displaying a neuroprotective effect against amyloid-β accumulation [25]. Although these motifs have been individually disclosed in potent MAO-B inhibitors, to our knowledge the structure–activity relationship of the combined arylpiperazine and cinnamyl pharmacophores into one scaffold as potential MAO-B inhibitors was not explored so far [26,27].

**Figure 2:**
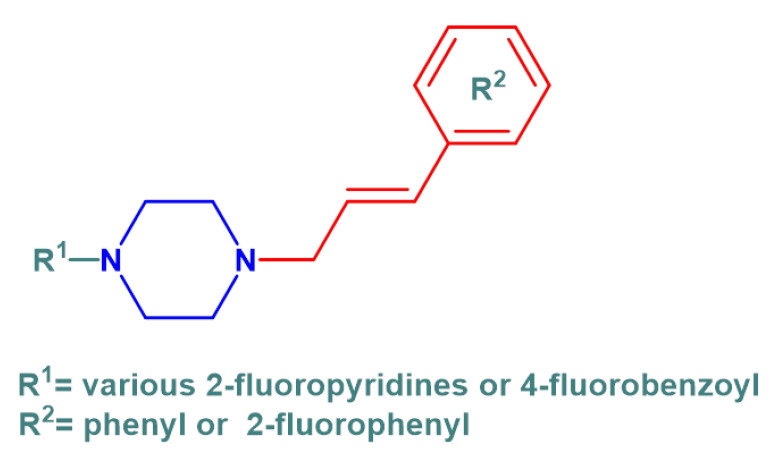
Design of fluorinated cinnamylpiperazines as potential MAO-B ligands. Fusion of the arylpiperazine (blue) and the cinnamyl pharmacophores (red) with different aryl fluorine-containing substituents.

We herein report on the organic synthesis of ten novel fluorinated cinnamylpiperazines as potentially active heterocyclic compounds targeting MAO-B (Figure 2). The primary idea of our concept relies on the hypothesis that the presence of the basic nitrogen group from 4-arylpiperazine and extended π-conjugated system from cinnamyl moiety in one scaffold can potentially enhance the MAO-B binding affinity via the formation of ionic interactions, hydrogen bonds and π-interactions with amino acid residues in the active site of the MAO-B. Moreover, besides the suggested enhancement of the metabolic stability and pharmacokinetic properties upon the insertion of fluorine into pharmacologically active molecules [28], in particular for PET development, the pyridyl and benzoyl groups substituted with fluorine in positions 2 and 4 would also favor the radiofluorination of these scaffolds via the aromatic nucleophilic substitution of known leaving groups [29,30,31]. Another crucial consideration is based on the exploitation of different isomers, if deemed to have an impact on the binding affinity and pharmacokinetic profile as previously reported by us [32] and others [33]. The lipophilicity described by the partition coefficient between octanol-phosphate buffer at physiological pH (Log*D*_7.4_) is also a key parameter on the development of BBB penetrant PET tracers, whereby compounds possessing a Log*D*_7.4_ in the range of 1–4 are more likely to passively diffuse through the BBB [34].

The binding affinities of the herein reported compounds were determined with a new in-house MAO-B binding affinity protocol using l-[^3^H]Deprenyl as radioligand. These results were used as pass-fail criteria for the further development of ^18^F-labeled PET tracers targeting MAO-B in the brain. To provide a deeper insight to our understanding regarding ligand-target interaction with our first generation of MAO-B compounds, molecular docking studies to the active site of human MAO-B were applied.

## 2. Results and Discussion

### 2.1. Chemistry

**Scheme 1:**
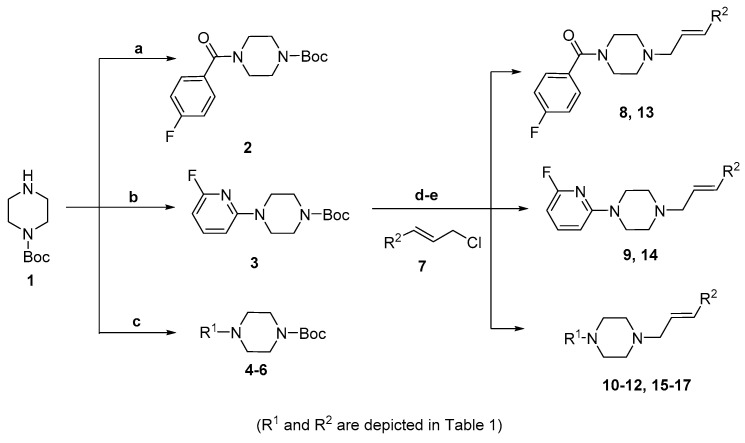
Synthetic route of fluorinated derivatives **8–17**. Reagents and conditions: (a) 4-fluorobenzoyl chloride, Et_3_N, CH_2_Cl_2_, 25 °C, 3 h, 96%; (b) 2,6-difluoropyridine, Et_3_N, *N,N*-dimethylformamide (DMF), reflux, 16 h, 63%; (c) Ar^1^Br, Pd(OAc)_2_, 2,2′-bis(diphenylphosphino)-1,1′-binaphthyl (BINAP), *t*-BuOK, dioxane, 110 °C, 19 h, 43–51%; (d) trifluoroacetic acid (TFA), 25 °C, 4 h, 96–98%; (e) **7**, Et_3_N, MeCN, 25 °C, 2.5–4.5 h, 43–59%.

The detailed synthetic route for the designed derivatives is depicted in Scheme 1 (for full characterization of the synthesized derivatives, see Supplementary Materials).

1-Boc-piperazine **1** was coupled with aromatic moieties by acylation, nucleophilic aromatic substitution and Buchwald–Hartwig reactions. Acylation of **1** with 4-fluorobenzoyl chloride afforded the intermediate **2** almost quantitatively [35]. The nucleophilic aromatic substitution of electron deficient 2,6-difluoropyridine with **1**, gave intermediate **3** in a moderate yield (Scheme 1) [36]. Intermediates **4**–**6** could not be synthesized via nucleophilic aromatic substitution due to low chemoselectivity of the bromo-substituted fluoropyridines. Instead, the Buchwald–Hartwig reaction was applied to obtain intermediates **4**–**6** in moderate yields (Scheme 1) [36]. TFA-mediated deprotection of Boc-carbamates **2**–**6** [37], followed by alkylation with cinnamic acid derivatives **7**, afforded the final compounds **8**–**17** in yields of about 43–59% in two steps (Scheme 1).

### 2.2. MAO-B Binding Affinity and LogD_7.4_ of Compounds ***8**–**17***

In order to evaluate the binding affinities of the herein synthesized MAO-B derivatives, we established a binding assay using l-[^3^H]Deprenyl as a radioligand (see Section 3.5). For the validation of our protocol, we reproduced the experimental conditions already reported [38] by using rat brain homogenates and l-Deprenyl as a reference compound. In our hands, a *K_D_* and *B_max_* values of 19.75 ± 4.60 nM and 2.29 ± 2.08 pmol/mg protein, respectively, were determined for l-[^3^H]Deprenyl (Table 1). A previous study [38], reporting saturation experiments performed on rat frontal cortex with [^3^H]Ro 19-6327, showed a similar *B_max_* value (3.45 pmol/mg protein) for l-Deprenyl while the IC_50_ value using the same radioligand (8 nM) indicated about two-fold higher affinity compared to our assay.

Unexpectedly, none of the herein reported MAO-B ligands **8**–**17** exhibited binding affinity in nM range, a prerequisite for PET applications. The Log*D*_7.4_ values calculated using Advanced Chemistry Development, Inc (ACD/labs^®^, version 12.0, Toronto, ON, Canada) revealed that all compounds possess optimal lipophilicities for a passive diffusion within the BBB (Table 1) [39].

### 2.3. Docking Studies

**Figure 3:**
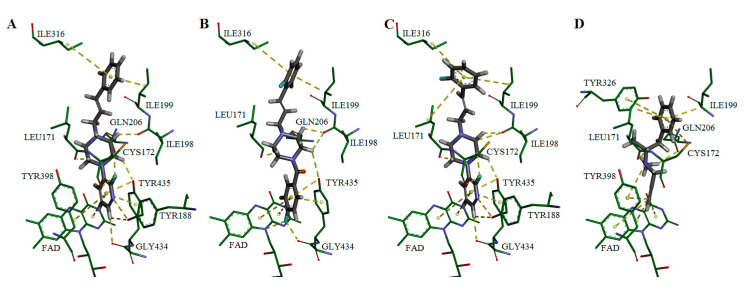
Docking poses of compound **12** (**A**), **13** (**B**), **17** (**C**) and l-Deprenyl (**D**) in the human MAO-B substrate binding site; ligand (grey) and key residues (green) are represented as stick models, while ligand interactions are shown as dashed lines (yellow).

Molecular docking studies were carried out to gain insight on the binding poses and intermolecular interactions of compounds **8**–**17** by using the X-ray crystallographic structure of human MAO-B (PDB ID: 2BYB, resolution: 2.2 Å) and the docking software GOLD 5.5. The fitness goldscores are summarized in Table 1 and representative docking poses of compounds **12**, **13**, **17** and l-Deprenyl as reference, are displayed in Figure 3. From what is known from previous studies, detrimental interactions in the binding pocket of MAO-B occur on (i) the substrate cavity composed of flavin adenine dinucleotide (FAD), PHE343, and four tyrosine residues, namely TYR188, TYR189, TYR435 and TYR398 and (ii) the entrance cavity involving LEU171, TYR 326, PHE168, ILE198 and ILE199 residues [28,40,41]. Depending on the nature of the inhibitor, the cavities remain separated or fused by rotation of ILE199.

Based on these premises, our docking studies revealed that compounds **8**–**17** share similar interactions with essential residues for MAO-B binding at both the substrate and entrance cavities in comparison to l-Deprenyl. The goldscores are in the same range for all proposed ligands (65–75, Table 1), and are superior to those obtained for l-Deprenyl (51, Table 1), thus pointing to a superior binding to MAO-B. While the arylpiperazine moiety of all herein synthesized ligands is located in the substrate cavity and is also stabilized by aromatic π-π interactions with TYR188, TYR435 and the FAD cofactor, the cinnamyl pharmacophores are oriented towards the hydrophobic entrance cavity and are stabilized by π-σ interactions with ILE199 and ILE316. We assume that the higher goldscores of compound **8**–**17** in comparison to l-Deprenyl are a result of the ability of fluorine to enhance the ligand affinity by participation in multipolar C-F⋯H-N and C-F⋯C=O interactions [42], especially with CYS172, ILE199, GLY434 or FAD for MAO-B [40,41]. In agreement with that, potent MAO-B cinnamyl-based inhibitors substituted with electron withdrawing groups such as fluorine, chlorine and trifluoromethyl group attached to the aromatic core were already disclosed [43]. Moreover, the piperazine nitrogen of compounds **8**–**17** would positively contribute to MAO-B binding upon the formation of water bridges and hydrogen bonding as reported for potent MAO-B benzhydrylpiperazine based inhibitors [26].

The greater discrepancies, in comparison to our reference l-Deprenyl, are related to the interaction of the phenyl group of **12** and the 2-fluorophenyl substituents for **13** and **17** with ILE316 and different TYR residues. The bipartite cavity plays an important role in the substrate–enzyme recognition and is therefore crucial for the design of specific reversible inhibitors [44]. We hypothesize that the interactions of compounds **8**–**17** in both cavities were not strong enough for an efficient binding to MAO-B, as reflected by the lack of binding affinity towards MAO-B in our assays (Table 1). Possible explanations for that might be addressed to (i) the additional interaction of the hydrophobic substituents and the residue ILE316, a common trend among the developed ligands (Figure 3) and (ii) the insertion of voluminous substituents near the substrate cavity.

It is worth noting that considerable care has to be taken when using the goldscore as a parameter for further ligand development as they are not directly correlated to the IC_50_ values. In particular, to low affinity binders the molecular modeling may be biased, thus having limited potential to forecast the affinity effects of structural modifications [45]. Moreover, some simplifications of the method which encompasses the exclusion of explicit water molecules and solvation and entropic effects might be taken into account in future analysis.

## 3. Materials and Methods

Unless stated otherwise all solvents were freshly distilled under argon prior to being used. All chemicals and reagents were purchased from commercially available sources and used without further purification. ^1^H and ^13^C NMR spectra were recorded on Bruker Avance III spectrometer, at 500 MHz for proton (^1^H) and at 126 MHz for carbon (^13^C). Chemical shifts are given in parts per million (ppm) from tetramethylsilane (TMS) as internal standard in CDCl_3_. 2D NMR spectra (HSQC) were recorded at 500 MHz. Coupling constants (*J*) are reported in hertz (Hz). Unless stated otherwise, all spectra were recorded at 25 °C. High-resolution mass spectra (HRMS) were recorded on a FT-ICR APEX II spectrometer (Bruker Daltonics; Bruker Corporation, Billerica, MA, USA) using electrospray ionization in positive ion mode (ESI+). All reactions were monitored by thin-layer chromatography (TLC). Flash and dry-column flash chromatography were carried out using silica gel (10–18 or 18–32 µm, ICN-Woelm). Melting points were obtained at a heating rate of 4 °C/min, and are uncorrected. IR spectra were recorded by using a Thermo Scientific Nicolet 6700 Fourier-transform spectrometer operated in the ATR mode. Structures of all new compounds were determined by methods of 1D, 2D NMR and IR spectroscopy. Structures of the final compounds were additionally confirmed by high-resolution mass spectrometry (HRMS).

### 3.1. tert-Butyl 4-(4-fluorobenzoyl)piperazine-1-carboxylate (***2***)

The synthesis of the fluorobenzoyl piperazine **2** was done as previously described with minor modifications [35]. To a solution of **1** (0.32 g, 1.7 mmol) and Et_3_N (1.1 equiv, 1.9 mmol) in CH_2_Cl_2_ (3 mL) 4-fluorobenzoyl chloride (1.5 equiv, 2.5 mmol,) was added at 0 °C. The reaction mixture was stirred at room temperature (r.t.) for 72 h. The resulting mixture was washed with 10% Na_2_CO_3_ solution (5 mL) then 10% HCl solution (5 mL), dried over anhydrous Na_2_SO_4_ and concentrated by rotary evaporator, yielding 96% of **2** as a viscous, yellow oil. The crude product was used in the next step without further purification; *R_f_* = 0.41 (SiO_2_; *n*-hexane/EtOAc = 8:2); ^1^H NMR (500 MHz, CDCl_3_): δ = 1.49 (s, 9H, COO(CH_3_)_3_), 3.41–3.47 (m, 4H, piperazine), 3.60–3.74 (m, 4H, piperazine), 7.12–7.46 (m, 4H, ArH) ppm; ^13^C NMR (126 MHz, CDCl_3_): δ = 28.5 (3C), 43.7 (2C), 46.1 (2C), 80.6, 115.8 (d, ^2^*J_CF_* = 21.8 Hz, 2C), 129.5 (d, ^3^*J_CF_* = 8.5 Hz, 2C), 131.5 (d, ^4^*J_CF_* = 2.2 Hz), 154.7, 163.6 (d, ^1^*J_CF_* = 250.3 Hz), 169.9 ppm.

### 3.2. tert-Butyl 4-(6-fluoropyridin-2-yl)piperazine-1-carboxylate (***3***)

The synthesis of the fluoropyridin piperazine **3** was done as previously described with minor modifications [36].To a solution of **1** (1.6 g, 8.69 mmol) in anhydrous DMF (33 mL), 2.6-difluoropyridine (1.0 equiv., 8.69 mmol) and Et_3_N (1.5 equiv., 13.08 mmol,) were added. The mixture was refluxed for 16 h. After cooling, the reaction mixture was quenched with saturated NaHCO_3_ solution (25 mL). Then, the mixture was diluted with water (75 mL) and extracted with EtOAc (3 × 60 mL). The combined organic layers were washed with water (2 × 50 mL) and brine (50 mL), dried over anhydrous Na_2_SO_4_ and concentrated by rotary evaporator. The crude product was purified by dry-column flash chromatography (SiO_2_; petrolether/EtOAc = 10:0 to 8:2) yielding 63% of **3** as a pale-yellow oil; *R_f_* = 0.50 (SiO_2_; *n*-hexane/EtOAc = 8:2); ^1^H NMR (500 MHz, CDCl_3_): δ = 1.48 (s, 9H, COO(CH_3_)_3_), 3.52 (s, 8H, piperazine), 6.18–6.20 (m, 1H, ArH), 6.40–6.42 (m, 1H, ArH), 7.53–7.55 (m, 1H, ArH) ppm; ^13^C NMR (126 MHz, CDCl_3_): δ = 28.5 (3C), 44.5 (2C), 44.9 (2C), 80.2, 96.5 (d, ^2^*J_CF_* = 37.6 Hz), 102.9 (d, ^4^*J_CF_* = 4.4 Hz), 142.1 (d, ^3^*J_CF_* = 8.3 Hz), 156.3, 158.2 (d, ^3^*J_CF_* = 15.7 Hz), 162.8 (d, ^1^*J_CF_* = 236.1 Hz) ppm.

### 3.3. General Procedure for the Synthesis of tert-Butyl 4-phenylpiperazine-1-carboxylate (***4**–**6***)

The phenylpiperazines **4**–**6** were synthesized according to standard protocols with minor modifications [36]. To a solution of **1** (l.58 g, 8.52 mmol) and aryl bromides (1.0 equiv., 8.52 mmol) in 1,4-dioxane (20 mL), *t*-BuONa (1.5 equiv., 12.79 mmol) was added. Nitrogen gas was purged through the reaction mixture for 5 min. Then, (±)-BINAP (0.06 equiv., 0.51 mmol) was added, followed by Pd(OAc)_2_ (0.06 equiv., 0.51 mmol). The reaction mixture was refluxed for 15 h. The mixture was cooled to r.t., diluted with H_2_O (20 mL), and extracted with EtOAc (3 × 150 mL). The combined organic layers were washed with brine (30 mL), dried over anhydrous Na_2_SO_4_ and concentrated by rotary evaporator. The crude product was purified by dry-column flash chromatography (SiO_2_; *n*-hexane/EtOAc = 10:0 to 8:2).

*Tert-butyl 4-(6-fluoropyridin-3-yl)piperazine-1-carboxylate* (**4**). Yield: 46%, pale-yellow oil; *R_f_* = 0.55 (SiO_2_; *n*-hexane/EtOAc = 8:2); ^1^H NMR (500 MHz, CDCl_3_): δ = 1.45 (s, 9H, COO(CH_3_)_3_), 3.04–3.06 (m, 4H, piperazine), 3.55–3.57 (m, 4H, piperazine) 6.79–6.81 (m, 1H, ArH), 7.32–7.34 (m, 1H, ArH), 7.55 (s, 1H, ArH) ppm; ^13^C NMR (126 MHz, CDCl_3_): δ = 28.5 (3C), 49.8 (4C), 80.2, 109.3 (d, ^2^*J_CF_* = 39.3 Hz), 130.1 (d, ^3^*J_CF_* = 7.5 Hz), 135.4 (d, ^3^*J_CF_* = 15.1 Hz), 145.6 (d, ^4^*J_CF_* = 4.3 Hz), 154.6, 158.1 (d, ^1^*J_CF_* = 232.8 Hz) ppm.

*Tert-butyl 4-(2-fluoropyridin-4-yl)piperazine-1-carboxylate* (**5**). Yield: 51%, pale-yellow oil; *R_f_* = 0.53 (SiO_2_; *n*-hexane/EtOAc = 8:2);^1^H NMR (500 MHz, CDCl_3_): δ = 1.49 (s, 9H, COO(CH_3_)_3_), 3.35–3.36 (m, 4H, piperazine), 3.56–3.58 (m, 4H, piperazine) 6.17 (s, 1H, ArH), 6.53–6.55 (m, 1H, ArH), 7.88 (d, *J* = 5 Hz, 1H, ArH,) ppm; ^13^C NMR (126 MHz, CDCl_3_): δ = 29.9 (3C), 47.5 (4C), 81.9, 93.4 (d, ^2^*J_CF_* = 43.0 Hz), 108.2 (d, ^4^*J_CF_* = 2.8 Hz), 149.2 (d, ^3^*J_CF_* = 18.5 Hz), 156.0, 160.3 (d, ^3^*J_CF_* = 11.3 Hz), 167.4 (d, ^1^*J_CF_* = 232.6 Hz) ppm.

*Tert-butyl 4-(2-fluoropyridin-3-yl)piperazine-1-carboxylate (***6***).* Yield: 43%, pale-yellow oil; *R_f_* = 0.56 (SiO_2_; *n*-hexane/EtOAc = 8:2); ^1^H NMR (500 MHz, CDCl_3_): δ = 1.49 (s, 9H, COO(CH_3_)_3_), 3.02–3.07 (m, 1H, piperazine), 3.51–3.56 (m, 6H, piperazine), 3.59–3.61 (m, 1H, piperazine), 6.63–6.67 (m, 1H, ArH), 7.47–7.51 (m, 1H, ArH), 8.19–8.20 (m, 1H, ArH) ppm; ^13^C NMR (126 MHz, CDCl_3_): δ = 29.5 (3C), 46.2 (4C), 81.0, 108.3, 114.7, 138.6, 149.0, 155.9, 160.4 ppm.

### 3.4. General Procedure for the Synthesis of Final Compounds ***8**–**17***

To a solution of **2**, **3** and **4**–**6** (1.4 mmol) 20% TFA (4 mL) in CH_2_Cl_2_ (1 mL) was added at 5 °C. The mixture was stirred at room temperature (r.t.) for 4 h. After the removal of the solvent by rotatory evaporation and the addition of saturated K_2_CO_3_ solution (10 mL), the mixture was extracted with CH_2_Cl_2_ (2 × 20 mL). The combined organic layers were washed with brine (10 mL), dried over anhydrous Na_2_SO_4_, filtered and concentrated by rotary evaporator. The crude product was dissolved in MeCN (1 mL) followed by the addition of Et_3_N (2.0 equiv., 10 mmol) and **7** (1.0 equiv., 5 mmol). The reaction mixture was stirred for 3.5–4.5 h at r.t. After completion of the reaction, the mixture was concentrated by rotary evaporator. The remaining residue was dissolved in CH_2_Cl_2_ (20 mL), washed with saturated K_2_CO_3_ solution (10 mL) and brine (10 mL), dried over anhydrous Na_2_SO_4_, filtered and concentrated by rotary evaporator. The crude product was purified by dry-column flash chromatography (SiO_2_; *n*-hexane/EtOAc = 6:4 to 3:7).

*(E)-(4-cinnamylpiperazin-1-yl)(4-fluorophenyl)methanone (***8***).* Yield: 55%, viscous, yellow oil; *R_f_* = 0.45 (SiO_2_; *n*-hexane/EtOAc = 4:6); IR (ATR): 3027, 2921, 2805, 1624, 1599, 1436, 1288, 1223, 1155, 1000, 848, 739, 693 cm^−1^*;* ^1^H NMR (500 MHz, CDCl_3_): δ = 2.49–2.56 (m, 4H, piperazine), 3.19 (d, *J* = 6.6 Hz, 2H, CH_2_), 3.46 (br. s, 2H piperazine), 3.79 (br. s, 2H, piperazine), 6.21–6.30 (m, 1H, CH=CH), 6.53 (d, *J* = 15.8 Hz, 1H, CH=CH), 7.07–7.10 (m, 2H, ArH), 7.20–7.27 (m, 1H, ArH), 7.28–7.35 (m, 2H, ArH), 7.35–7.43 (m, 4H, ArH) ppm; ^13^C NMR (126 MHz, CDCl_3_): δ = 42.4 (2C), 47.9, 53.1, 60.9, 115.6 (d, ^2^*J_CF_* = 21.7 Hz, 2C), 125.8, 126.4 (2C), 127.8, 128.7 (2C), 129.4 (d, ^3^*J_CF_* = 8.8 Hz, 2C), 131.9 (d, ^4^*J_CF_* = 3.7 Hz, 2C), 133.7, 136.7, 163.5 (d, ^1^*J_CF_* = 249.7 Hz), 169.5 ppm; HRMS-ESI: calcd. for C_20_H_22_FN_2_O [M + H]^+^ = 325.17162; found 325.17835.

*1-Cinnamyl-4-(6-fluoropyridin-2-yl)piperazine (***9***).* Yield: 49%, viscous, yellow oil; *R_f_* = 0.47 (SiO_2_; *n*-hexane/EtOAc = 4:6); IR (ATR): 3028, 2946, 2827, 1615, 1560, 1481, 1443, 1265, 1220, 1134, 995, 772, 736, 690 cm^−1^; ^1^H NMR (500 MHz, CDCl_3_): δ = 2.51 (t, *J* = 5.0 Hz, 4H, piperazine), 3.12–3.13 (m, 2H, CH_2_), 3.49 (t, *J* = 5.0 Hz, 4H, piperazine), 6.08 (dd, *J* = 7.8, 2.9 Hz, 1H, CH=CH), 6.22 (dt, *J* = 15.8, 6.8 Hz, 1H, CH=CH), 6.33 (dd, *J* = 8.2, 2.5 Hz, 1H, ArH), 6.47 (d, *J* = 15.9 Hz, 1H, ArH), 7.14–7.18 (m, 1H, ArH), 7.24 (t, *J* = 7.6 Hz, 2H, ArH), 7.31 (d, *J* = 7.3 Hz, 2H, ArH), 7.44 (q, *J* = 8.2 Hz, 1H, ArH) ppm; ^13^C NMR (126 MHz, CDCl_3_): δ = 45.1 (2C), 52.9 (2C), 61.2, 96.1 (d, ^2^*J_CF_* = 37.5 Hz), 102.8 (d, ^4^*J_CF_* = 4.3 Hz), 126.2, 126.5 (2C), 127.7, 128.7 (2C), 133.5, 136.9, 141.9 (d, ^3^*J_CF_* = 8.3 Hz), 158.5 (d, ^3^*J_CF_* = 15.5 Hz), 162.9 (d, ^1^*J_CF_* = 235.4 Hz) ppm; HRMS-ESI: calcd. for C_18_H_21_FN_3_ [M + H]^+^ = 298.17195; found 298.17986.

*1-Cinnamyl-4-(6-fluoropyridin-3-yl)piperazine (***10***).* Yield: 55%, viscous, yellow oil; *R_f_* = 0.43 (SiO_2_; *n*-hexane/EtOAc = 4:6); IR (ATR): 3027, 2939, 282, 2771, 1586, 1494, 1453, 1350, 1252, 1143, 834, 742 cm^−1^; ^1^H NMR (500 MHz, CDCl_3_): δ = 2.67 (t, *J* = 5.0 Hz, 4H, piperazine), 3.17 (t, *J* = 5.0 Hz, 4H, piperazine), 3.21–3.23 (m, 2H, CH_2_), 6.28 (dt, *J* = 15.8, 6.8 Hz, 1H, CH=CH), 6.56 (d, *J* = 15.8 Hz, 1H, CH=CH), 6.81(dd, *J* = 8.9, 3.5 Hz, 1H, ArH), 7.22–7.25 (m, 1H, ArH), 7.30–7.35 (m, 3H, ArH), 7.38–7.40 (m, 2H, ArH), 7.78–7.79 (m, 1H, ArH) ppm; ^13^C NMR (126 MHz, CDCl_3_): δ = 49.5 (2C), 53.9 (2C), 61.0, 109.16 (d, ^2^*J_CF_* = 39.2 Hz), 126.1, 126.4 (2C), 128.7 (2C), 129.2 (d, ^3^*J_CF_* = 7.3 Hz), 133.5, 134.7 (d, ^3^*J_CF_* = 15.0 Hz), 136.8, 145.6 (d, ^4^*J_CF_* = 3.9 Hz), 157.8 (d, ^1^*J_CF_* = 232.2 Hz) ppm; HRMS-ESI: calcd. for C_18_H_21_FN_3_ [M + H]^+^ = 298.17195; found 298.17621.

*1-Cinnamyl-4-(2-fluoropyridin-4-yl)piperazine (***11***).* Yield: 49%, viscous, yellow oil; *R_f_* = 0.44 (SiO_2_; *n*-hexane/EtOAc = 4:6); IR (ATR): 3027, 2924, 2850, 1613, 1549, 1501, 1449, 1265, 1192, 995, 820, 744 cm^−1^; ^1^H NMR (500 MHz, CDCl_3_): δ = 2.61 (t, *J* = 5.0 Hz, 4H, piperazine), 3.20–3.22 (m, 2H, CH_2_), 3.37 (t, *J* = 5.0 Hz, 4H, piperazine), 6.17–6.18 (m, 1H, CH=CH), 6.27 (dt, *J* = 15.9, 6.8 Hz, 1H, CH=CH), 6.53–6.57 (m, 2H, ArH), 7.23–7.26 (m, 1H, ArH), 7.31–7.34 (m, 2H, ArH), 7.39 (d, *J* = 7.3 Hz, 2H, ArH), 7.87 (d, *J* = 5.0 Hz,1H, ArH) ppm; ^13^C NMR (126 MHz, CDCl_3_): δ = 46.3 (2C), 52.5 (2C), 61.0, 91.8 (d, ^2^*J_CF_* = 42.7 Hz), 106.7 (d, ^4^*J_CF_* = 2.0 Hz), 125.9, 126.4 (2C), 127.8, 128.7 (2C), 133.7, 136.8, 147.6 (d, ^3^*J_CF_* = 18.6 Hz), 159.0 (d, ^3^*J_CF_* = 11.6 Hz), 165.9 (d, ^1^*J_CF_* = 232.5 Hz) ppm; HRMS-ESI: calcd. for C_18_H_21_FN_3_ [M + H]^+^ = 298.17195; found 298.17728.

*1-Cinnamyl-4-(2-fluoropyridin-3-yl)piperazine (***12***).* Yield: 52%*,* viscous, yellow oil; *R_f_* = 0.46 (SiO_2_; *n*-hexane/EtOAc = 4:6); IR (ATR): 3026, 2929, 2819, 1702, 1598, 1567, 1454, 1237, 1142, 970, 798, 747, 695 cm^−1^; ^1^H NMR (500 MHz, CDCl_3_): δ = 2.64–2.66 (m, 4H, piperazine), 3.10–3.12 (m, 4H, piperazine), 3.19 (d, *J* = 6.8 Hz, 2H, CH_2_), 6.24 (dt, *J* = 15.9, 6.8 Hz, 1H, CH=CH), 6.53 (d, *J* = 15.8 Hz, 1H, CH=CH), 7.02–7.05 (m, 1H, ArH), 7.17–7.22 (m, 2H, ArH), 7.27 (t, *J* = 7.6 Hz, 2H, ArH), 7.34 (d, *J* = 7.7 Hz, 2H, ArH), 7.70 (d, *J* = 4.8Hz, 1H, ArH) ppm; ^13^C NMR (126 MHz, CDCl_3_): δ = 50.1, 50.1, 53.2 (2C), 61.2, 121.9 (d, ^4^*J_CF_* = 4.2 Hz), 126.3, 126.5 (2C), 127.4 (d, ^3^*J_CF_* = 5.0 Hz), 127.7, 128.7 (2C), 133.7, 135.5 (d, ^2^*J_CF_* = 23.2 Hz), 137.0, 138.4 (d, ^3^*J_CF_* = 14.5 Hz), 156.1 (d, ^1^*J_CF_* = 239.3 Hz) ppm; HRMS-ESI: calcd. for C_18_H_21_FN_3_ [M + H] ^+^ = 298.17195; found 298.17234.

*(E)-(4-Fluorophenyl)(4-(3-(2-fluorophenyl)allyl)piperazin-1-yl)methanone (***13***).* Yield: 45%, viscous, yellow oil; *R_f_* = 0.47 (SiO_2_; *n*-hexane/EtOAc = 4:6); IR (ATR): 3064, 2927, 2810,1635, 1457, 1434, 1284, 1229, 1156, 1002, 849, 760 cm^−1^; ^1^H NMR (500 MHz, CDCl_3_): δ = 2.48–2.54 (m, 4H, piperazine), 3.21 (d, *J* = 6.6 Hz, 2H, CH_2_), 3.46 (br. s, 2H, piperazine), 3.78 (br. s, 2H, piperazine), 6.31 (dt, *J* = 16.1, 6.7 Hz, 1H, CH=CH), 6.68 (d, *J* = 16.0 Hz, 1H, CH=CH), 6.98–7.04 (m, 1H, ArH), 7.04–7.12 (m, 3H, ArH), 7.18–7.22 (m, 1H, ArH), 7.38–7.45 (m, 3H, ArH) ppm; ^13^C NMR (126 MHz, CDCl_3_): δ = 42.4 (2C), 47.9 (2C), 53.1, 61.2, 115.3–116.0 (m, 3C), 124.2 (d, ^4^*J_CF-1_* = 3.6 Hz), 124.5 (d, ^2^*J_CF-1_* = 11.9 Hz), 125.98 (d, ^4^*J_CF-1_* = 3.6 Hz), 127.41 (d, ^3^*J_CF-1_* = 3.9 Hz), 128.5 (d, ^3^*J_CF-1_* = 4.6 Hz), 129.02 (d, ^3^*J_CF-1_* = 8.4 Hz), 129.5 (d, ^3^*J_CF-2_* = 8.7 Hz, 2C), 131.8 (d, ^4^*J_CF-2_* = 3.5 Hz), 160.2 (d, ^1^*J_CF-1_* = 248.9 Hz), 163.5 (d, ^1^*J_CF-2_* = 249.9 Hz), 169.5 ppm; HRMS-ESI: calcd. for C_20_H_21_F_2_N_2_O [M + H]^+^ = 343.16220; found 343.16892.

*(E)-1-(3-(2-Fluorophenyl)allyl)-4-(6-fluoropyridin-2-yl)piperazine (***14***).* Yield: 58%, viscous, yellow oil; *R_f_* = 0.46 (SiO_2_; *n*-hexane/EtOAc = 4:6); IR (ATR): 3040, 2933, 2815, 1614, 1563, 1486, 1443, 1266, 1230, 997, 778, 758 cm^−1^; ^1^H NMR (500 MHz, CDCl_3_): δ = 2.60 (t, *J* = 5.0 Hz, 4H, piperazine), 3.22–3.24 (m, 2H, CH_2_), 3.57 (t, *J* = 5.0 Hz, 4H, piperazine), 6.61 (dd, *J* = 7.7, 2.9 Hz, 1H, CH=CH), 6.34–6.42 (m, 2H, CH=CH and ArH), 6.71 (d, *J* = 16.0 Hz, 1H), 7.01–7.05 (m, 1H, ArH), 7.08–7.11 (m, 1H, ArH), 7.18–7.23 (m, 1H, ArH), 7.47 (td, *J* = 8.6, 7.7 Hz, 1H, ArH), 7.52 (q, *J* = 8.2 Hz, 1H, ArH) ppm; ^13^C NMR (126 MHz, CDCl_3_): δ = 45.1 (2C), 52.9 (2C), 61.4, 96.1 (d, ^2^*J_CF-2_* = 37.7 Hz), 107.7 (d, ^4^*J_CF-2_* = 3.1 Hz), 115.8 (d, ^2^*J_CF-1_* = 22.3 Hz), 124.2 (d, ^4^*J_CF-1_* = 3.6 Hz), 124.7 (d, ^2^*J_CF-1_* = 12.4 Hz), 125.8 (d, ^3^*J_CF-1_* = 3.7 Hz), 127.43 (d, ^3^*J_CF-1_* = 3.9 Hz), 128.8–129.1 (m, 2C, 141.9 (d, ^3^*J_CF-2_* = 8.3 Hz), 158.5 (d, ^3^*J_CF-2_* = 16.1 Hz), 160.3 (d, ^1^*J_CF-1_* = 249.4 Hz), 162.9 (d, ^1^*J_CF-2_* = 235.3 Hz) ppm; HRMS-ESI: calcd. for C_18_H_20_F_2_N_3_ [M + H]^+^ = 316.16253; found 316.16811.

*(E)-1-(3-(2-Fluorophenyl)allyl)-4-(6-fluoropyridin-3-yl)piperazine (***15***).* Yield: 50%, viscous, yellow oil; *R_f_* = 0.44 (SiO_2_; *n*-hexane/EtOAc = 4:6); IR (ATR): 3041, 2927, 2821, 1732, 1584, 1493, 1455, 1394,1252, 1144, 1003, 834, 757 cm^−1^; ^1^H NMR (500 MHz, CDCl_3_): δ = 2.69 (t, *J* = 5.0 Hz, 4H, piperazine), 3.20 (t, *J* = 5.0 Hz, 4H, piperazine), 3.26 (d, *J* =6.9 Hz, 2H, CH_2_), 6.37 (dt, *J* = 16.0, 6.8 Hz, 1H, CH=CH), 6.73 (d, *J* = 16.0 Hz, 1H, CH=CH), 6.81–6.83 (m, 1H, ArH), 7.01–7.11 (m, 2H, ArH), 7.19–7.23 (m, 1H, ArH), 7.33–7.36 (m, 1H, ArH), 7.45–7.49 (m, 1H, ArH), 7.79–7.80 (m, 1H. ArH) ppm; ^13^C NMR (126 MHz, CDCl_3_): δ = 49.6 (2C), 53.0 (2C), 61.3, 109.2 (d, ^1^*J_CF-2_* = 39.2 Hz), 115.8 (d, ^2^*J_CF-1_* = 22.0 Hz), 124.2 (d, ^4^*J_CF-1_* = 3.5 Hz), 124.6 (d, ^2^*J_CF-1_* = 12.1 Hz), 125.9 (d, ^3^*J_CF-2_* = 3.6 Hz), 127.4 (d, ^3^*J_CF-1_* = 3.7 Hz), 128.8 (d, ^4^*J_CF-1_* = 3.7 Hz), 129.0 (d, ^3^*J_CF-1_*= 8.2 Hz), 129.4 (d, ^3^*J_CF-1_* = 7.4 Hz), 134.8 (d, ^3^*J_CF-2_* = 15.0 Hz), 145.6 (d, ^4^*J_CF-2_* = 4.2 Hz), 158.1 (d, ^1^*J_CF-1_*= 289.5 Hz), 160.1 (d, ^1^*J_CF-2_* = 306.0 Hz) ppm; HRMS-ESI: calcd. for C_18_H_20_F_2_N_3_ [M + H]^+^ = 316.16253; found 316.16992.

*(E)-1-(3-(2-Fluorophenyl)allyl)-4-(2-fluoropyridin-4-yl)piperazine (***16***).* Yield: 59%, viscous, yellow oil; *R_f_* = 0.45 (SiO_2_; *n*-hexane/EtOAc = 4:6); IR (ATR): 3041, 2924, 2849, 2818, 1613, 1549, 1452, 1265, 1193, 996, 820, 758 cm^−1^; ^1^H NMR (500 MHz, CDCl_3_): δ = 2.62 (t, *J* = 5.0 Hz, 4H, piperazine), 3.23 (d, *J* = 6.8 Hz, 2H, CH_2_), 3.37(t, *J* = 5.0 Hz, 4H, piperazine), 6.17–6.18 (m, 1H, CH=CH), 6.34 (dt, *J* = 16.0, 6.8 Hz, 1H, CH=CH), 6.53–6.55 (m, 1H, ArH), 6.71 (d, *J* = 15.9 Hz, 1H, ArH), 6.98–7.06 (m, 1H, ArH), 7.09 (t, *J* = 7.7 Hz, 1H), 7.19–7.23 (m, 1H, ArH), 7.44–7.48 (m, 1H, ArH), 7.87(d, *J* = 6.0 Hz, 1H, ArH) ppm; ^13^C NMR (126 MHz, CDCl_3_): δ = 46.3 (2C), 52.5 (2C), 61.2, 91.8 (d, ^2^*J_CF-2_* = 42.7 Hz), 106.7 (d, ^4^*J_CF-2_* = 2.0 Hz), 115.9 (d, ^2^*J_CF-1_* = 22.0 Hz), 124.2 (d, ^3^*J_CF-1_* = 3.6 Hz), 124.6 (d, ^2^*J_CF-1_* = 12.3 Hz), 126.0 (d, ^4^*J_CF-1_* = 3.6 Hz), 127.4 (d, ^3^*J_CF-1_* = 4.4 Hz), 128.6 (d, ^4^*J_CF-1_* = 4.5 Hz), 129.1 (d, ^3^*J_CF-1_* = 8.3 Hz), 147.6 (d, ^3^*J_CF-2_* = 18.8 Hz), 159.1 (d, ^3^*J_CF-2_* = 11.4 Hz), 159.4 (d, ^1^*J_CF-1_* = 252.1 Hz), 166.0 (d, ^1^*J_CF-2_* = 232.3 Hz) ppm; HRMS-ESI: calcd. for C_18_H_20_F_2_N_3_ [M + H]^+^ 316.16253; found 316.16590.

*(E)-1-(3-(2-Fluorophenyl)allyl)-4-(2-fluoropyridin-3-yl)piperazine (***17***).* Yield: 43%, viscous, yellow oil; *R_f_* = 0.44 (SiO_2_; *n*-hexane/EtOAc = 4:6); IR (ATR): 3061, 2936, 2820, 17001, 1569, 1487, 1454, 1324, 1143, 973, 796, 756 cm^−1^; ^1^H NMR (500 MHz, CDCl_3_): δ = 2.64–2.65 (m, 4H, piperazine), 3.10–3.12 (m, 4H, piperazine), 3.20 (d, *J* = 6.8 Hz, 2H, CH_2_), 6.31 (dt, *J* = 16.0, 6.8 Hz, 1H, CH=CH), 6.67 (d, *J* = 6.0 Hz, 1H, CH=CH), 6.95–6.99 (m, 1H, ArH), 7.02–7.05 (m, 2H, ArH), 7.12–7.21 (m, 2H, ArH), 7.41(t, *J* = 7.6 Hz, 1H, ArH), 7.68–7.69 (m, 1H, ArH) ppm; ^13^C NMR (126 MHz, CDCl_3_): δ = 50.1, 50.1, 53.2 (2C), 61.4, 115.9 (d, ^2^*J_CF-2_* = 22.3 Hz), 122.0 (d, ^4^*J_CF-2_* = 4.0 Hz), 124.3 (d, ^4^*J_CF-1_* = 3.6 Hz), 124.8 (d, ^2^*J_CF-1_*= 12.3 Hz), 125.9 (d, ^3^*J_CF-1_* = 3.8 Hz), 127.4 (d, ^3^*J_CF-1_* = 4.9 Hz), 127.5 (d, ^3^*J_CF-1_* = 3.8 Hz), 128.9–129.1 (m, 2C), 135.5 (d, ^2^*J_CF-1_* = 23.1 Hz), 138.5 (d, ^3^*J_CF-2_* = 14.5 Hz), 156.1 (d, ^1^*J_CF-1_* = 239.5 Hz), 160.3 (d, ^1^*J_CF-2_* = 249.1 Hz) ppm; HRMS-ESI: calcd. for C_18_H_20_F_2_N_3_ [M + H]^+^ = 316.16253; found 316.16345.

### 3.5. In Vitro Binding Experiments

The affinity of the synthesized derivatives towards MAO-B was determined in radioligand competition binding assays. The assays were performed using rat brain membrane homogenates and the MAO-B-specific radioligand l-[^3^H]Deprenyl (obtained from Novandi Chemistry AB, NT1063). Membrane suspension was incubated with 2 nM l-[^3^H]deprenyl and various concentrations of the test compound in 50 mM Tris−HCl, pH 7.4 buffer containing 120 mM NaCl, 5 mM KCl, 2 mM MgCl_2_, at r.t. for 60 min. Non-specific binding was determined in the presence of 10 μM of rasagiline. The reaction was terminated by rapid filtration using Whatman GF/B glass-fiber filters, pre-soaked in 0.3% polyethyleneimine, and a 48-channel harvester (Biomedical Research and Development Laboratories, Gaithersburg, MD, USA) followed by washing four times with ice-cold TRIS-HCl buffer. Filter-bound radioactivity was quantified by liquid scintillation counting. The results represent two single experiments, each performed in triplicate. The determination of the *K*_D_ value of *L*-Deprenyl was acquired with homologous competition with the non-radioactive ligand in the range (10^−11^–10^−5^ M). The data were analyzed with GraphPad Prism, Version 4.1 (GraphPad Inc., La Jolla, CA, USA).

### 3.6. Docking Stimulations

Molecular docking studies were carried out using GOLD (Genetic Optimization for Ligand Docking) 5.5 program from Cambridge Crystallographic Data Center (CCDC, Cambridge, UK). GOLD uses a genetic algorithm for docking ligands into protein binding sites to explore the full range of ligand conformational flexibility with partial flexibility of the active site of the protein [46]. The X-ray crystallographic protein structure of human MAO-B in complex with deprenyl (PDB ID: 2BYB) was considered for the purpose of docking stimulation. Among the several other crystal structures in the Protein Data Bank (PDB), this structure was particularly selected due to the high resolution of 2.2 Å. The MAO-B protein was prepared by using the protein preparation wizard tool implemented in the GOLD software that removes all water molecules and adds hydrogen atoms to the protein structure. After removing deprenyl from the protein structure, an active site of radius 15 Å was defined considering the phenolic oxygen atom TYR435 in the substrate cavity. The ligand preparation was carried out in CambridgeSoft Chem3D 17.0 program from PerkinElmer (Waltham, MA, USA). The energy of each compound was minimized by using the MM2 force field method. Ten docking runs were performed per structure and the early termination step was activated if the first three poses have a root-mean-square deviation (RMSD) value of less than 1.5 Å, other parameters were set as default. After docking, the individual binding poses of each compound were observed and their molecular interactions within the active site were evaluated. The program Discovery Studio 2017 from BIOVIA^®^ (San Diego, CA, USA) was used to visualize the key aspects of the docking results from GOLD.

## 4. Conclusions

In summary, a simple and scalable synthetic route to obtain potential MAO-B ligands was herein reported. Starting from commercially available compounds, the fluorinated cinnamylpiperazines derivatives **8**–**17** were obtained in moderate overall yields. A new and robust binding affinity protocol using l-[^3^H]Deprenyl as radioligand was successfully developed. Our preliminary efforts to develop potent MAO-B ligands for further use as PET tracers revealed that none of the fluorinated derivatives exhibited sufficient binding affinity. Based on docking studies, we assume that voluminous substituents in the substrate cavity might have a detrimental impact on the binding affinities. Hence, not only might the design of highly affine MAO-B ligands rely on the stabilization of the ligands in both entrance and substrate cavities, but also additional requirements on the entrance cavity might be crucial. Based on these findings, further derivatization using fluorinated cinnamylpiperazines scaffolds will not be considered for the development of new MAO-B PET imaging agents.

## Figures and Tables

**Table 1:** Fitness goldscore, MAO-B binding affinities (IC_50_ in µM) and calculated log*D*_7.4_ of the synthesized compounds **8**–**17**.

	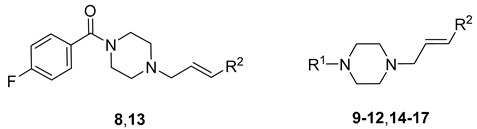				
Compound	R^1^	R^2^	Fitness Goldscore ^a^	IC_50_ (µM) ^b^	log*D*_cal_
**8**	/	phenyl	72.6	>10	3.55
**9**	6-(2-fluoropyridyl)	phenyl	67.3	>10	3.53
**10**	5-(2-fluoropyridyl)	phenyl	68.1	>10	2.56
**11**	4-(2-fluoropyridyl)	phenyl	66.4	>10	2.85
**12**	3-(2-fluoropyridyl)	phenyl	70.0	>10	2.50
**13**	/	2-fluorophenyl	75.8	>10	3.50
**14**	6-(2-fluoropyridyl)	2-fluorophenyl	70.4	>10	3.47
**15**	5-(2-fluoropyridyl)	2-fluorophenyl	70.5	>10	2.50
**16**	4-(2-fluoropyridyl)	2-fluorophenyl	67.0	>10	2.80
**17**	3-(2-fluoropyridyl)	2-fluorophenyl	70.7	>10	2.44
**l-Deprenyl**			51.3	19.75 ± 4.60 ^c^	2.31

^a^ Higher scores indicate more favorable binding. ^b^ IC_50_ values represent two independent experiments done in triplicate. ^c^ *K*_D_ (nM) determined in two independent homologous binding experiments preformed in triplicate. *B_max_* = 2.29 ± 2.08 pmol/mg protein.

## Supplementary Materials

The following are available online. The full experimental procedure for compound **1** and for the cinnamic acid derivatives **7**. NMR spectra of final compounds **8**–**17**.
