# Supplementary material for: Newly Synthesized Fluorinated Cinnamylpiperazines Possessing Low In Vitro MAO-B Binding
Source: Molecules. 2020 Oct 26;25(21):4941. doi: 10.3390/molecules25214941 (PMC7663645; doi:10.3390/molecules25214941)
Supplement: Supplementary file 1 [file molecules-25-04941-s001.pdf]

Supporting Information

Newly synthesized fluorinated cinnamylpiperazines possessing low *in vitro* MAO-B binding

Ivana I. Jevtić[1†], Thu Hang Lai[2,3†], Jelena Z. Penjišević[1], Sladjana Dukić-Stefanović[2], Deana B. Andrić[4], Peter Brust[2], Sladjana V. Kostić-Rajačić[1], Rodrigo Teodoro[2†*]

[1] ICTM-Department of Chemistry, University of Belgrade, Njegoševa 12, 11000 Belgrade, Serbia; ivana.jevtic@ihtm.bg.ac.rs; jelena.penjisevic@ihtm.bg.ac.rs; sladjana.kostic@ihtm.bg.ac.rs

[2] Helmholtz-Zentrum Dresden-Rossendorf, Institute of Radiopharmaceutical Cancer Research, Department of Neuroradiopharmaceuticals, Research site Leipzig, Permoserstraße 15, 04318 Leipzig, Germany; t.lai@hzdr.de; r.teodoro@hzdr.de; s.dukic-stefanovic@hzdr.de; p.brust@hzdr.de

[3] ROTOP Pharmaka GmbH, Department of Research and Development, Dresden, Germany; t.lai@hzdr.de

[4] Faculty of Chemistry, University of Belgrade, Studentski trg 12-16, 11000 Belgrade, Serbia; deanad@chem.bg.ac.rs

[†] These authors contributed equally to this work

* Correspondence: r.teodoro@hzdr.de; Tel.: +49 341 234 179 4636

1. GENERAL

Unless stated otherwise all solvents were freshly distilled under argon prior to being used. All reagents were purchased from commercially available sources, and were used without further purification. ^{1}H and ^{13}C NMR spectra were recorded on Bruker Avance™ III spectrometer, at 500 MHz for the proton (^{1}H) and at 126 MHz for the carbon (^{13}C). Chemical shifts are given in parts per million from tetramethylsilane (TMS) as internal standard in $CDCl_3$. 2D NMR spectra (HSQC) were recorded at 500 MHz Coupling constants (*J*) are reported in Hz. Unless stated otherwise all spectra were recorded at 25 °C. High resolution mass spectra (HRMS) were recorded on a FT-ICR APEX II spectrometer (Bruker Daltonics; Bruker Corporation, Billerica, MA, USA) using electrospray ionization (ESI) in positive ion mode. All reactions were monitored by thin layer chromatography (TLC). Flash and dry-column flash chromatography were carried out using silica gel (10–18 or 18-32 μm, ICN-Woelm). Melting points were obtained at a heating rate of 4 °C/min, and are uncorrected. IR spectra were recorded by using a Thermo Scientific Nicolet 6700 Fourier-transform spectrometer operated in the ATR mode. Structures of all new compounds were determined by methods of 1D, 2D NMR and IR spectroscopy. Structures of the final compounds were additionally confirmed by high resolution mass spectrometry (HRMS).

2. SYNTHESIS

*2.1. Synthesis of tert-butyl piperazine-1-carboxylate (**1**)*[1]

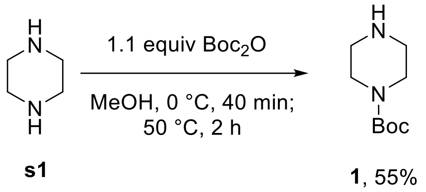

To a solution of piperazine hydrochloride **s1** (5.0 g, 58 mmol) in 20 mL of MeOH a solution of Boc_2O (1.1 equiv., 64 mmol) in 15 mL of MeOH was added drop wise over a period of 30 min at 0 °C and under argon atmosphere. The reaction mixture was heated to 50 °C for 2 h. After completion of the reaction, the 2 M solution of MeOLi (1.1 equiv.) in MeOH was added, and after stirring for additional 15 min, the mixture was concentrated by the rotary evaporator. The remaining solid was dissolved in 4 x 15 mL of EtOAc, to remove *bis* adduct. The combined organic layers were washed with 4 x 15 mL of water to remove the excess of piperazine, dried over the anhydrous Na_2SO_4 and concentrated by rotary evaporator yielding 55% of **1** as a white solid; mp 70 °C; R_f = 0.5 (SiO_2; *n*-hexane/EtOAc = 8:2); ^{1}H NMR (500 MHz, $CDCl_3$): δ = 1.45 (s, 9H, $COOCH_3$), 1.82 (s, 1H, NH), 2.79-2.81 (m, 4H, piperazine), 3.37-3.39 (m, 4H, piperazine) ppm; ^{13}C NMR (126 MHz, $CDCl_3$): δ = 28.3, 45.8, 79.5, 154.7 ppm.

*2.2. Synthesis of cinnamic acid derivatives (**7**)*

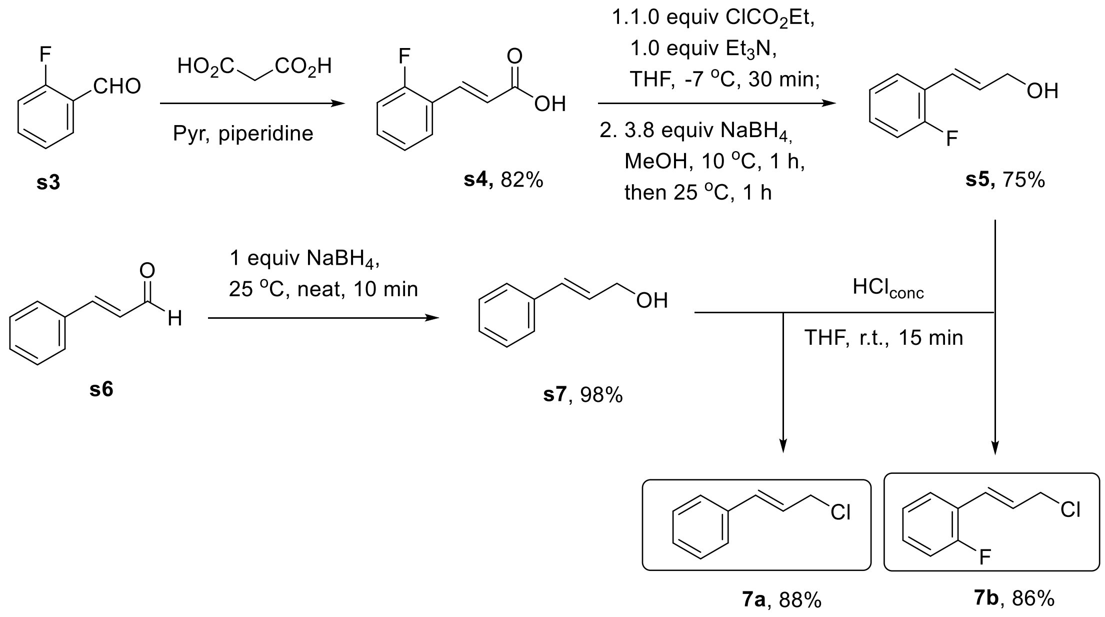

*2.2.1. (E)-3-(2-fluorophenyl)acrylic acid (**s4**)*[2]

Malonic acid (1.2 g, 1.2 mmol) is dissolved in 180 mL of dry pyridine. Then, 2-fluoro benzaldehyde **s3** (0.8 equiv., 1.0 mmol) and piperidine (0.08 equiv., 0.1 mmol) were added. The mixture is heated to 90 ° C for 1h. When the mixture is cooled, it is poured into mixture of ice and HCl. The crude product was recrystallized from EtOH/H_2O (2:1), yielding 82% of **s4** as a yellow solid; mp 177 °C; R_f = 0.2 (SiO_2; CH_2Cl_2/MeOH/Et_3N = 9:1:0.5); ^{1}H NMR (500 MHz, $CDCl_3$): δ = 6.56-6.59 (m, 1H, CH=CH), 7.22-7.28 (m, 2H, ArH), 7.43-7.46 (m, 1H, ArH), 7.62-7.65 (m, 1H, CH=CH), 7.79-7.81 (m, 1H, ArH) ppm; ^{13}C NMR (126 MHz, $CDCl_3$): δ = 116.4 (d, $^2J_{CF}$ = 20.0 Hz), 122.1 (d, $^4J_{CF}$ = 3.1 Hz), 122.2 (d, $^2J_{CF}$ = 19.8 Hz), 125.3 (d, $^4J_{CF}$ = 3.1 Hz), 132.6 (d, $^3J_{CF}$ = 8.1 Hz), 136.0 (d, $^3J_{CF}$ = 7.9 Hz), 161.8 (d, $^1J_{CF}$ = 252.1 Hz), 167.7 ppm.

*2.2.2. Synthesis of (E)-3-(2-fluorophenyl)prop-2-en-1-ol (**s5**)*[2]

To a solution of **s4** (3.3 g, 19.9 mmol) and Et_3N (1.0 equiv., 19.9 mmol) in dry THF (30 mL) $ClCO_2Et$ (1.0 equiv., 19.9 mmol) was added dropwise at –7 °C. After being stirred for an additional 30 min, the reaction mixture was allowed to warm to 10 °C and powdered $NaBH_4$ (3.8 equiv., 76 mmol) was added in one portion. Then dried MeOH (12 mL) was added dropwise over one hour at 10 °C. After being stirred at r.t. for additional one hour, the reaction mixture was poured into saturated NH_4Cl solution (200 mL) and extracted with CH_2Cl_2 (3 x 50 mL). The combined organic layers were dried over anhydrous Na_2SO_4 and concentrated. The crude product was purified by flash column chromatography (CH_2Cl_2/MeOH = 99:1) yielding 75% of **s5** as a pale-yellow oil; R_f = 0.3 (SiO_2; *n*-hexane/EtOAc = 8:2); ^{1}H NMR (500 MHz, $CDCl_3$): δ = 4.33(d, *J* = 5.6Hz, 2H, CH_2), 6.44(dt, *J* = 16.2, 5.6 Hz, 1H, CH=CH), 6.76 (d, *J* = 16Hz, 1H, CH=CH), 7.00-7.10 (m, 2H, ArH), 7.18-7.26 (m, 1H, ArH), 7.42-7.46 m (1H, ArH) ppm; ^{13}C NMR (126 MHz, $CDCl_3$): δ = 65.3, 117.3 (d, $^2J_{CF}$ = 20.0 Hz), 124.9 (d, $^3J_{CF}$ = 8.1 Hz), 125.7 (d, $^4J_{CF}$ = 3.1 Hz), 126.1(d, $^2J_{CF}$ = 20.0 Hz), 129.2 (d, $^3J_{CF}$ = 8.1 Hz), 130.5 (d, $^3J_{CF}$ = 7.9 Hz), 132.8 (d, $^4J_{CF}$ = 2.9 Hz), 160.8 (d, $^1J_{CF}$ = 252.1 Hz) ppm.

*2.2.3. Synthesis of (E)-3-phenylprop-2-en-1-ol (**s7**)*[3]

A mixture of **s6** (5.0 g, 37.8 mmol), and $NaBH_4$ (1.0 equiv., 37.8 mmol) was pestle for 10 min until full conversion. Then, the mixture was quenched with a saturated aqueous solution of $NaHCO_3$, followed by extraction of the alcohol with CH_2Cl_2 (3 x 20 mL). The combined organic layers were washed with brine (15 mL), dried over the Na_2SO_4 and concentrated by rotary evaporator yielding 98% of **s7** as a pale-yellow oil; R_f = 0.3 (SiO_2; *n*-hexane/EtOAc = 8:2); ^{1}H NMR (500 MHz, $CDCl_3$): δ = 1.91 (s, 1H, OH), 4.30 (dd, *J* = 5.7, 1.6 Hz, 2H, CH_2), 6.34 (dt, *J* = 15.9, 5.7 Hz, 1H, CH=CH), 6.60 (dt, *J* = 16.0, 1.6 Hz, 1H, CH=CH), 7.23 (tt, *J* = 6.5, 1.4 Hz, 1H, ArH), 7.26 – 7.33 (m, 2H, ArH), 7.33 – 7.38 (m, 2H, ArH) ppm; ^{13}C NMR (126 MHz, $CDCl_3$): δ = 65.2, 128.0, 129.2, 130.1, 130.2, 132.6, 138.3 ppm.

*2.2.4. General procedure for the synthesis of synthesis of **7a** and **7b**[4]*

To a solution of **s5** or **s7** (15 mmol) in THF (1 mL) concentrated HCl (3 mL) was added in 3 portions. The reaction mixture was stirred for 15 min at r.t. After full conversion, 5 mL of water was added and the mixture was extracted with CH_2Cl_2 (2 x 10 mL). The combined organic layers were washed with saturated K_2CO_3 solution (15 mL), then brine (15 mL), dried over the Na_2SO_4 and concentrated by rotary evaporator. The crude product was used in the next step without further purification.

*22.4.1. (E)-(3-chloroprop-1-en-1-yl)benzene (**7a**)*

Yield: 88 %, colorless oil; R_f = 0.6 (SiO_2; *n*-hexane/EtOAc = 9:1); ^{1}H NMR (500 MHz, $CDCl_3$): δ = 4.23-4.24 (m, 2H, CH_2), 6.31 (dt, *J* = 15.6, 7.3 Hz, 1H, CH=CH), 6.65 (d, *J* = 15.6 Hz, 1H, CH=CH), 7.24-7.28 (m, 1H, ArH), 7.33 (t, *J* = 7.4Hz, 2H, ArH), 7.39 (d, *J* = 7.3 Hz, 2H, ArH) ppm; ^{13}C NMR (126 MHz, $CDCl_3$): δ = 47.04, 126.5, 128.3, 129.9, 130.3, 135,8, 137.5 ppm..

*2.2.4.2. (E)-1-(3-chloroprop-1-en-1-yl)-2-fluorobenzene (**7b**)*

Yield: 86 %, colorless oil; R_f = 0.6 (SiO_2; *n*-hexane/EtOAc = 9:1); ^{1}H NMR (500 MHz, $CDCl_3$): δ = 4.26 (dd, *J* = 7.1, 1.2 Hz, 2H, CH_2), 6.42 (dt, *J* = 15.8, 7.1 Hz, 1H, CH=CH), 6.82 (d, *J* = 15.8 Hz, 1H, CH=CH), 7.02–7.16 (m, 2H, ArH), 7.22 – 7.32 (m, 1H, ArH), 7.46 (td, *J* = 7.7, 1.8 Hz, 1H, ArH) ppm; ^{13}C NMR (126 MHz, $CDCl_3$): δ = 45.5, 115.94 (d, $^2J_{CF}$ = 21.9 Hz), 123.84 (d, $^2J_{CF}$ = 11.9 Hz), 124.32 (d, $^4J_{CF}$ = 3.5 Hz), 126.71 (d, $^3J_{CF}$ = 3.8 Hz), 127.60 (d, $^3J_{CF}$ = 5.4 Hz), 127.84 (d, $^4J_{CF}$ = 3.7 Hz), 129.7 (d, $^3J_{CF}$ = 8.3 Hz), 160.51 (d, $^1J_{CF}$ = 250.5 Hz) ppm.

3. SPECTRA OF THE FINAL COMPOUNDS 8-17

^{1}H NMR (500 MHz, $CDCl_3$)

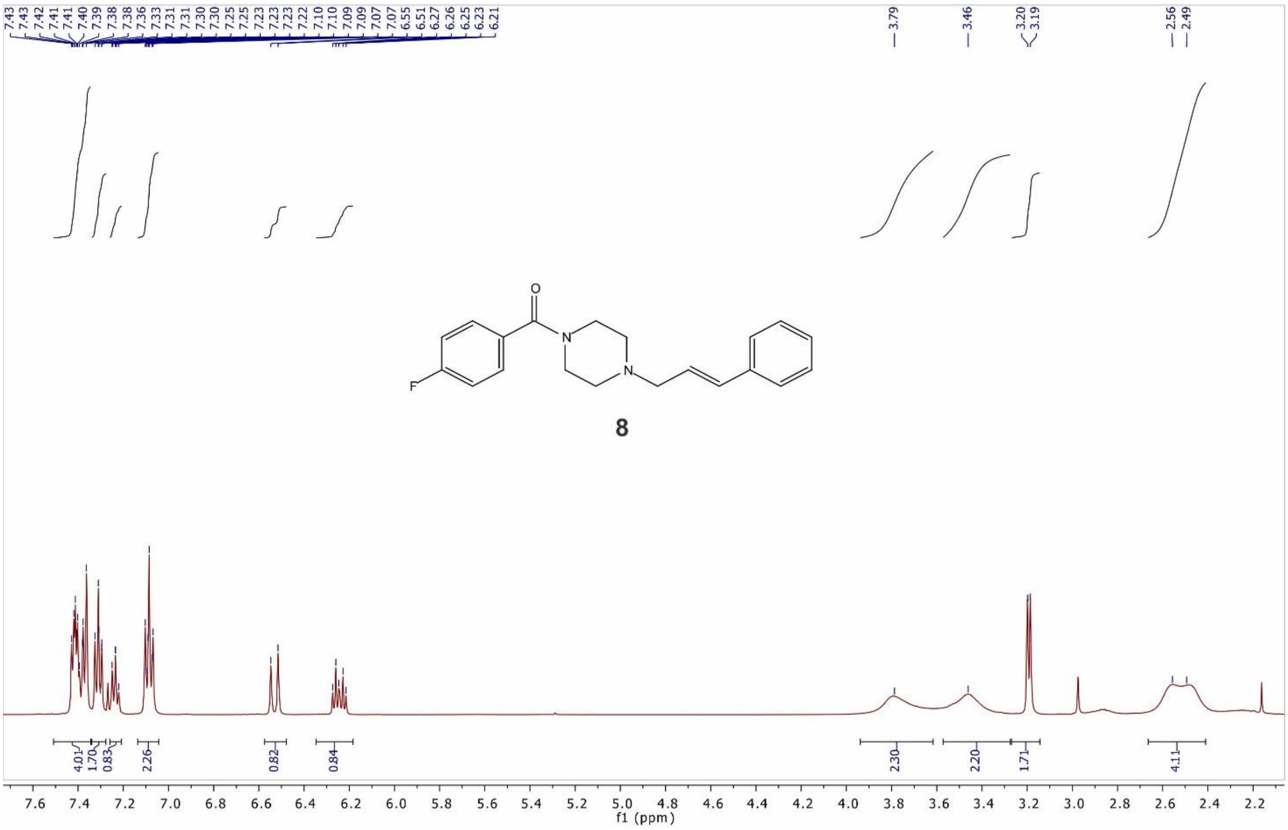

^{13}C NMR (126 MHz, $CDCl_3$)

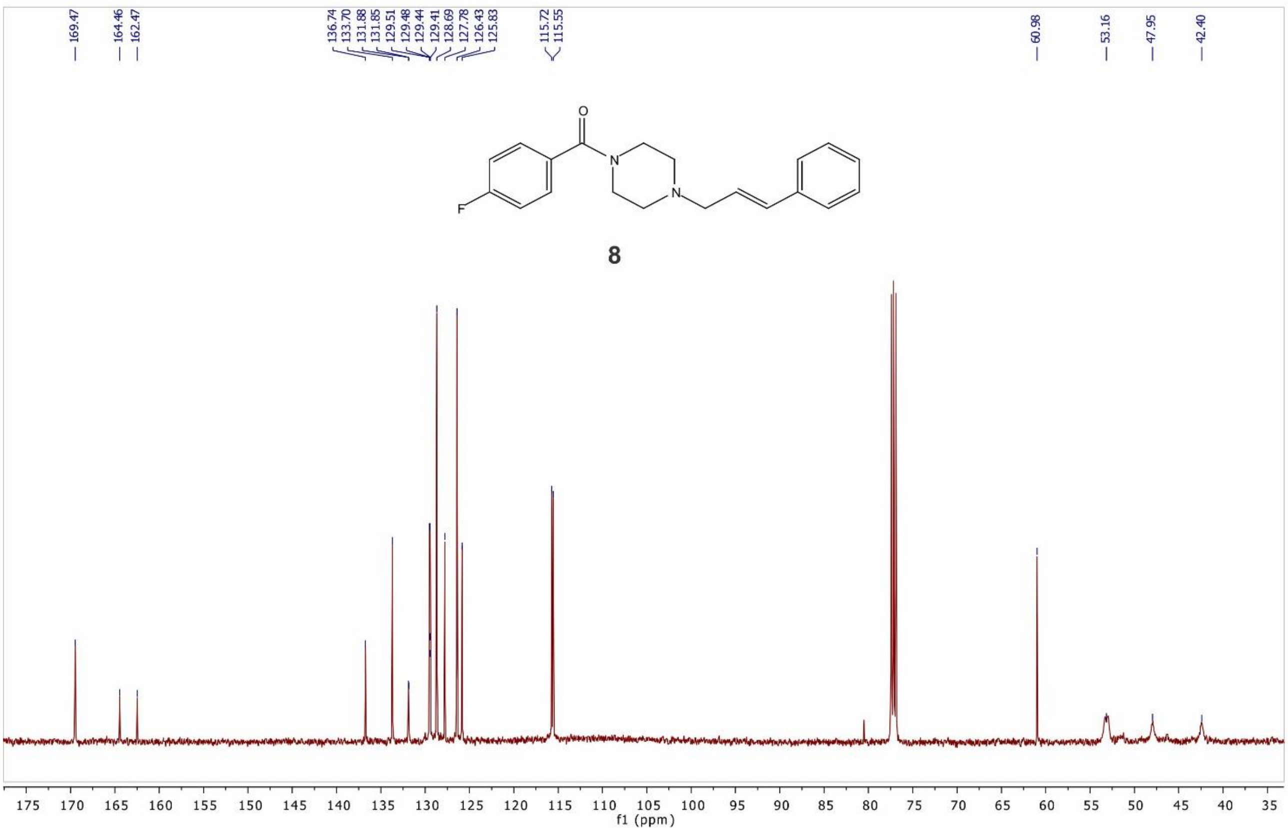

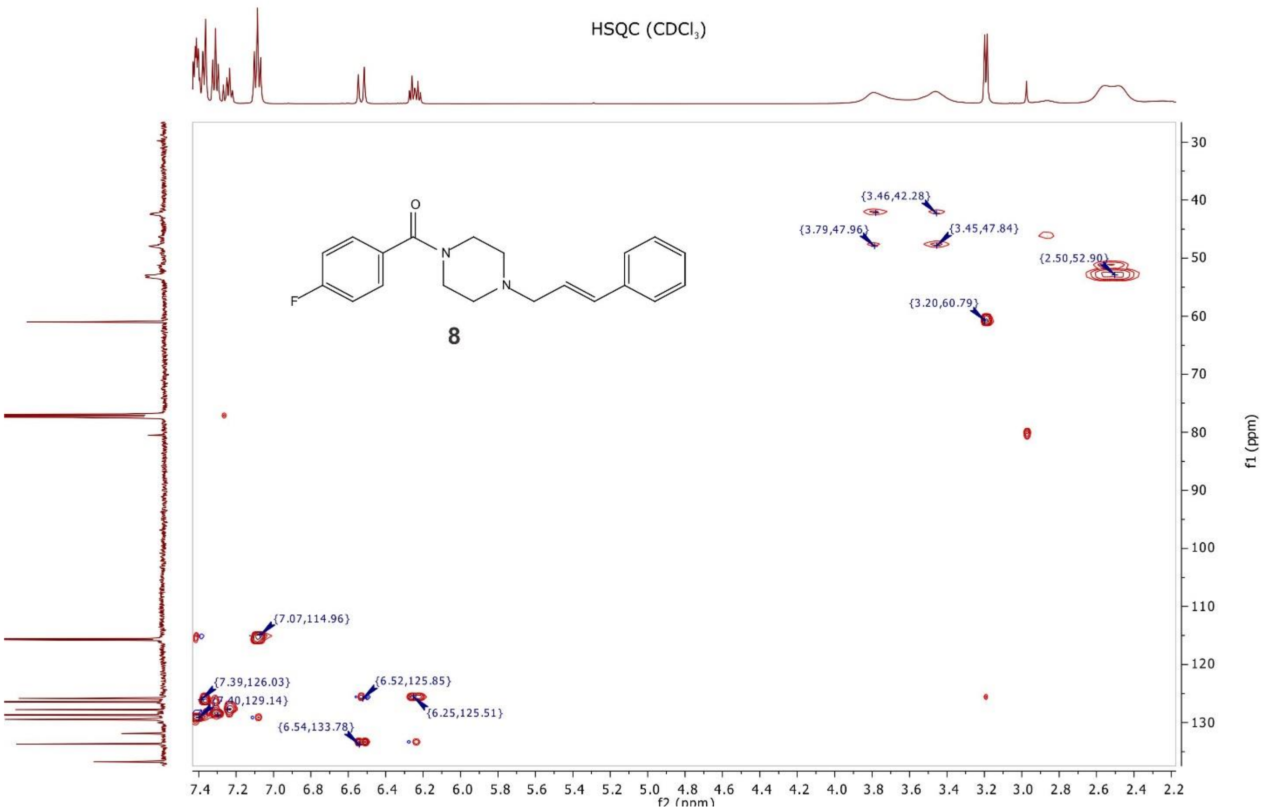
HSQC ($CDCl_3$)
8
f2 (ppm)
f1 (ppm)

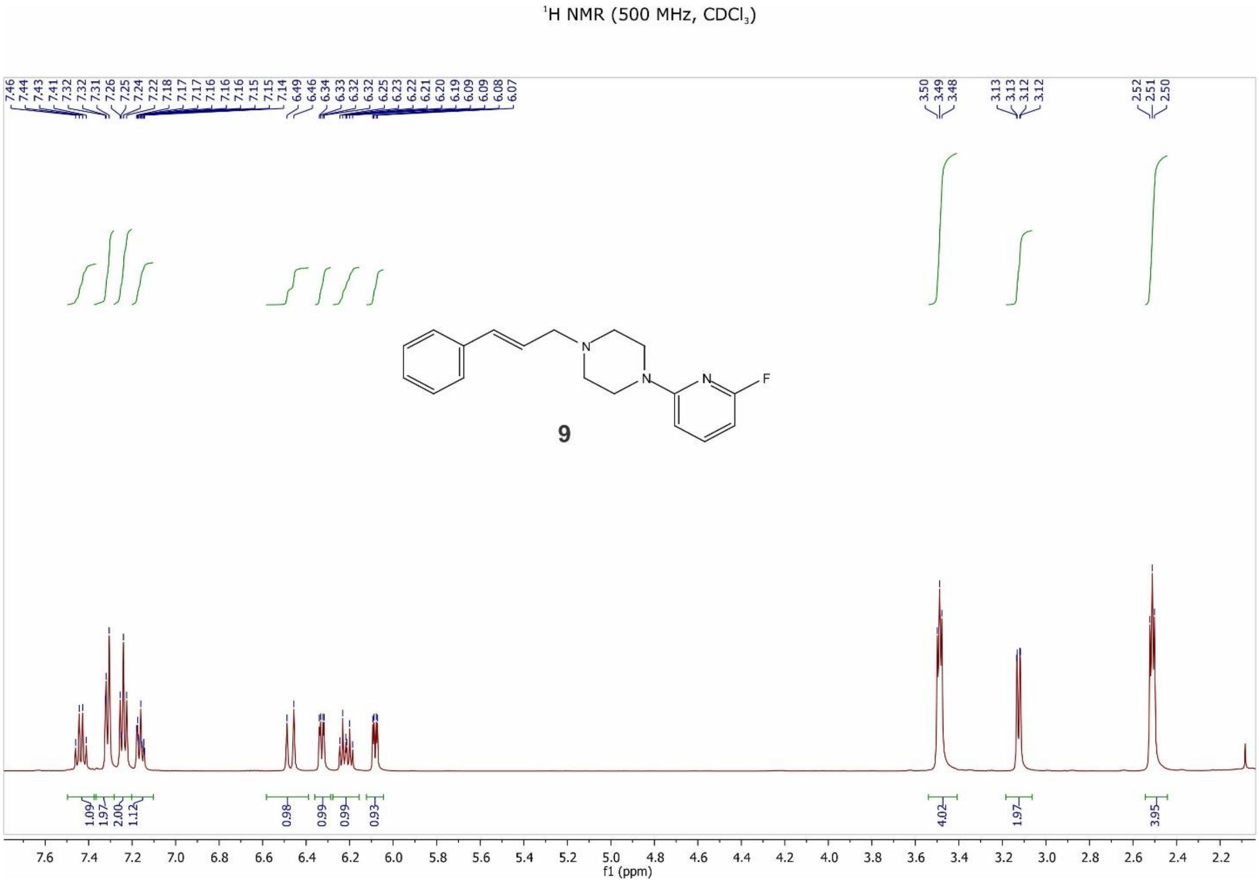
^{1}H NMR (500 MHz, $CDCl_3$)
9
f1 (ppm)

^{13}C NMR (126 MHz, $CDCl_3$)

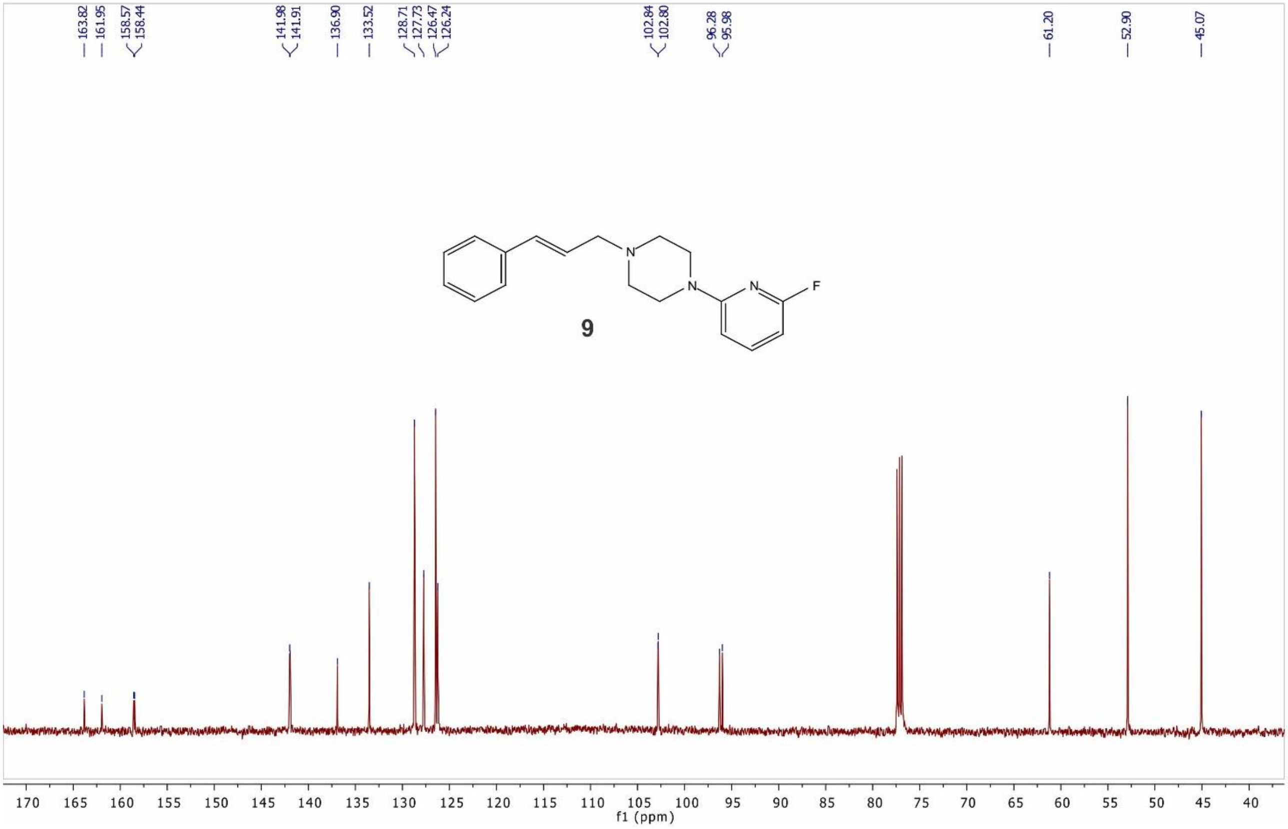

APT (126 MHz, $CDCl_3$)

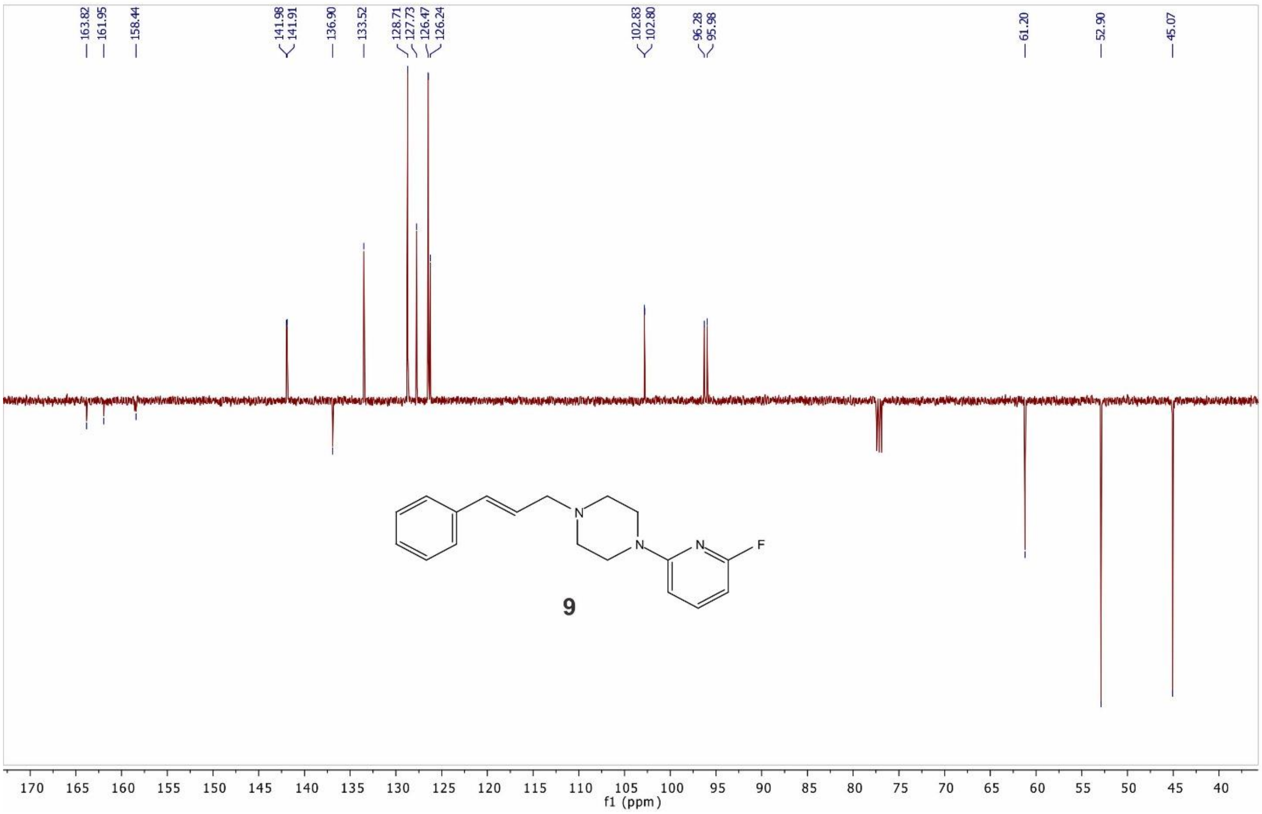

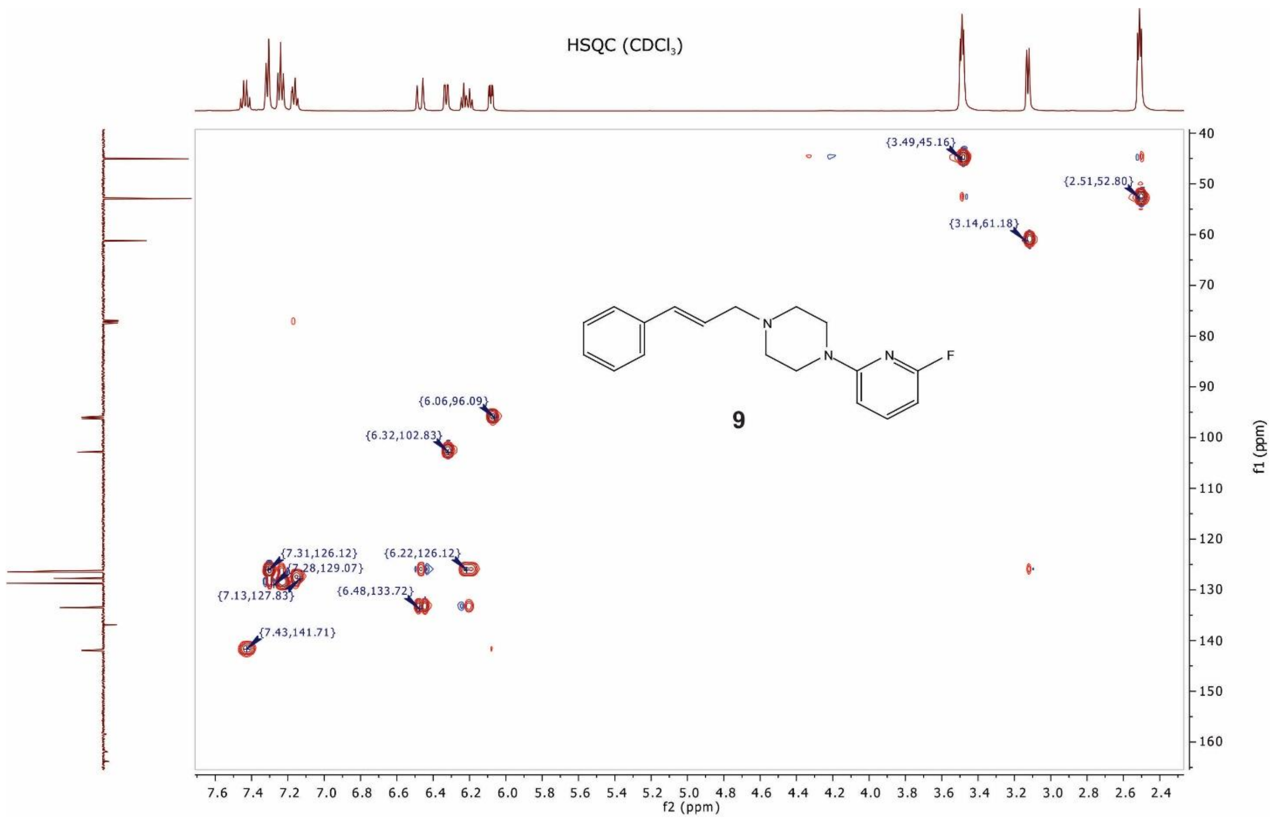

^1H NMR (500 MHz, $CDCl_3$)

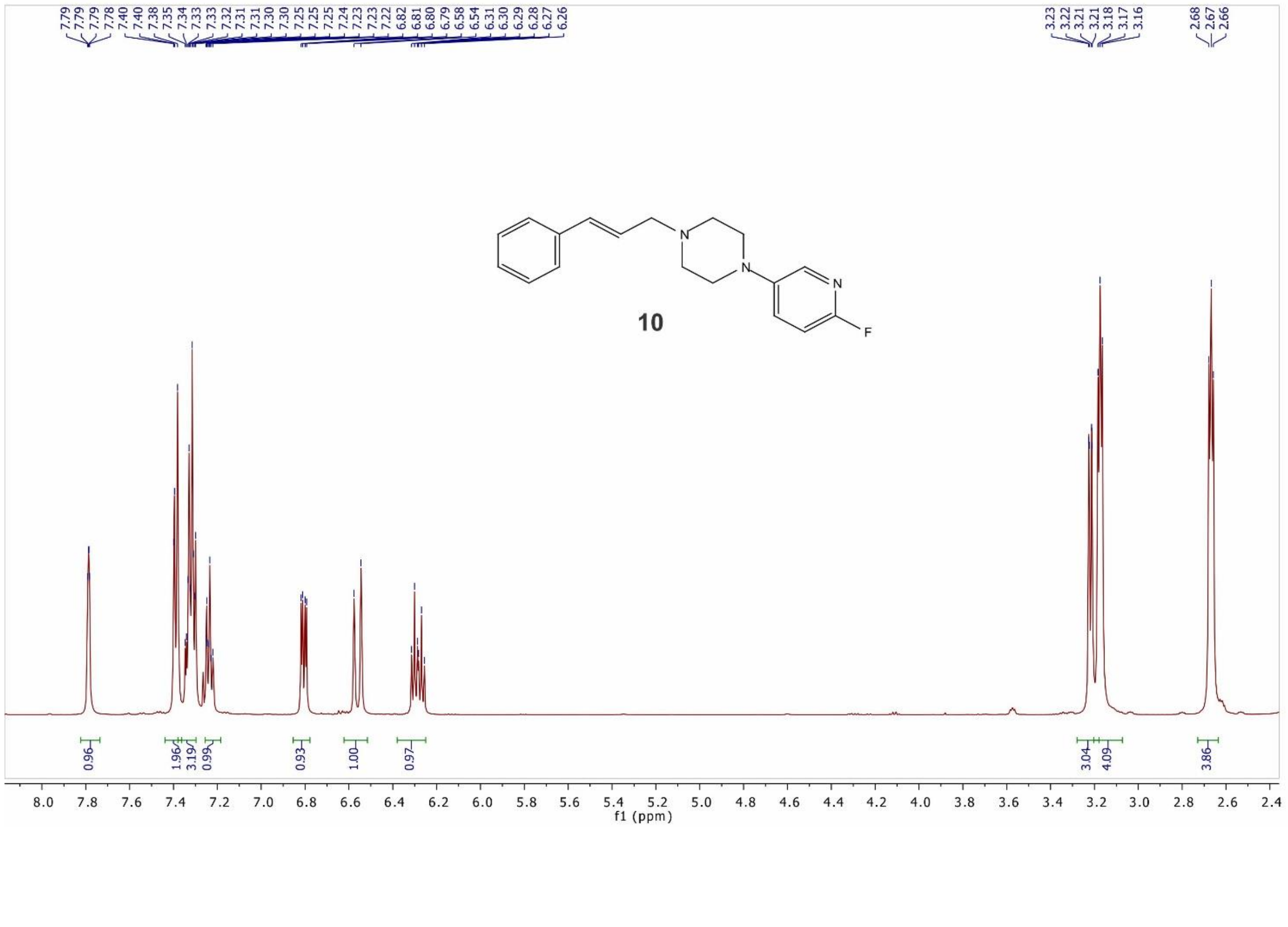

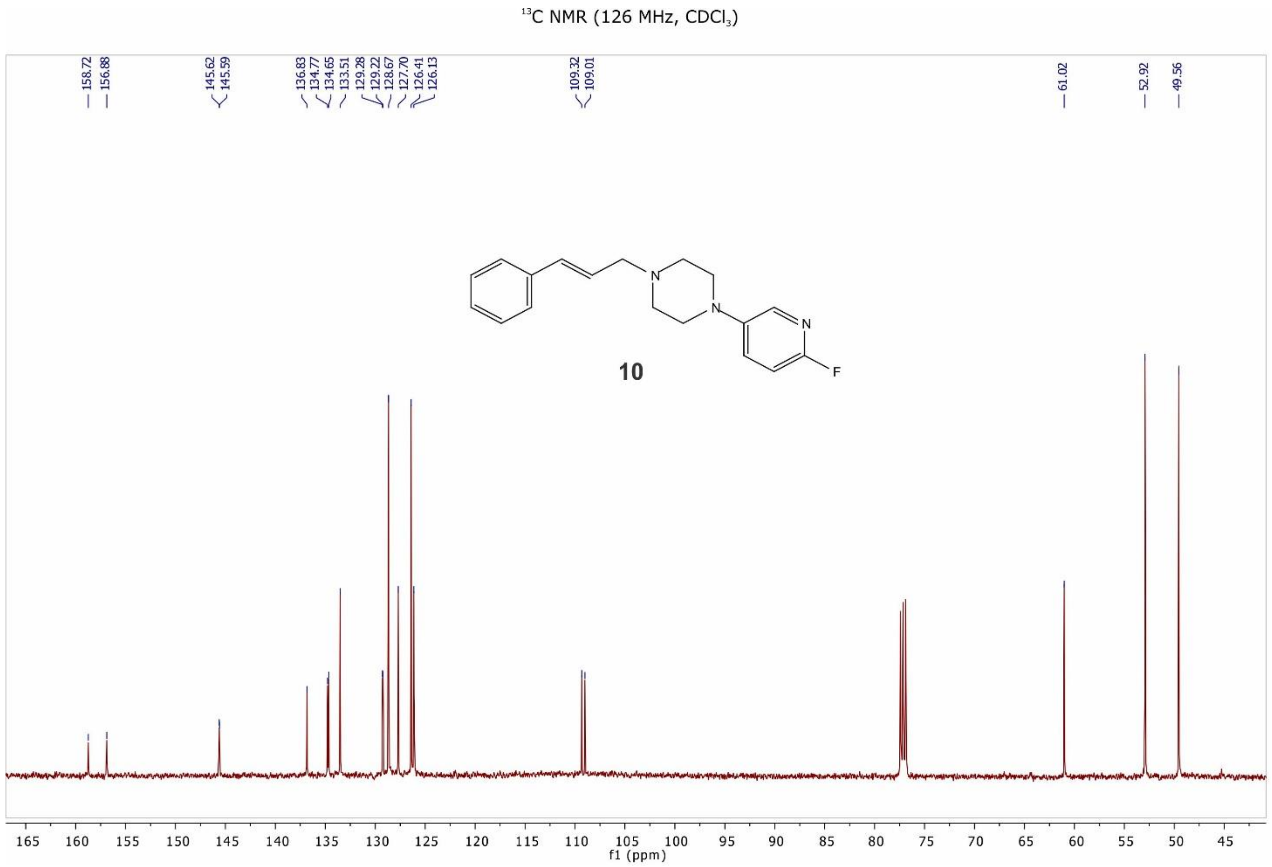
13C NMR (126 MHz, CDCl3)
158.72
156.88
145.62
145.59
136.83
134.77
134.65
133.51
129.28
129.22
128.67
127.70
126.41
126.13
109.32
109.01
61.02
52.92
49.56
N
N
N
F
10
f1 (ppm)

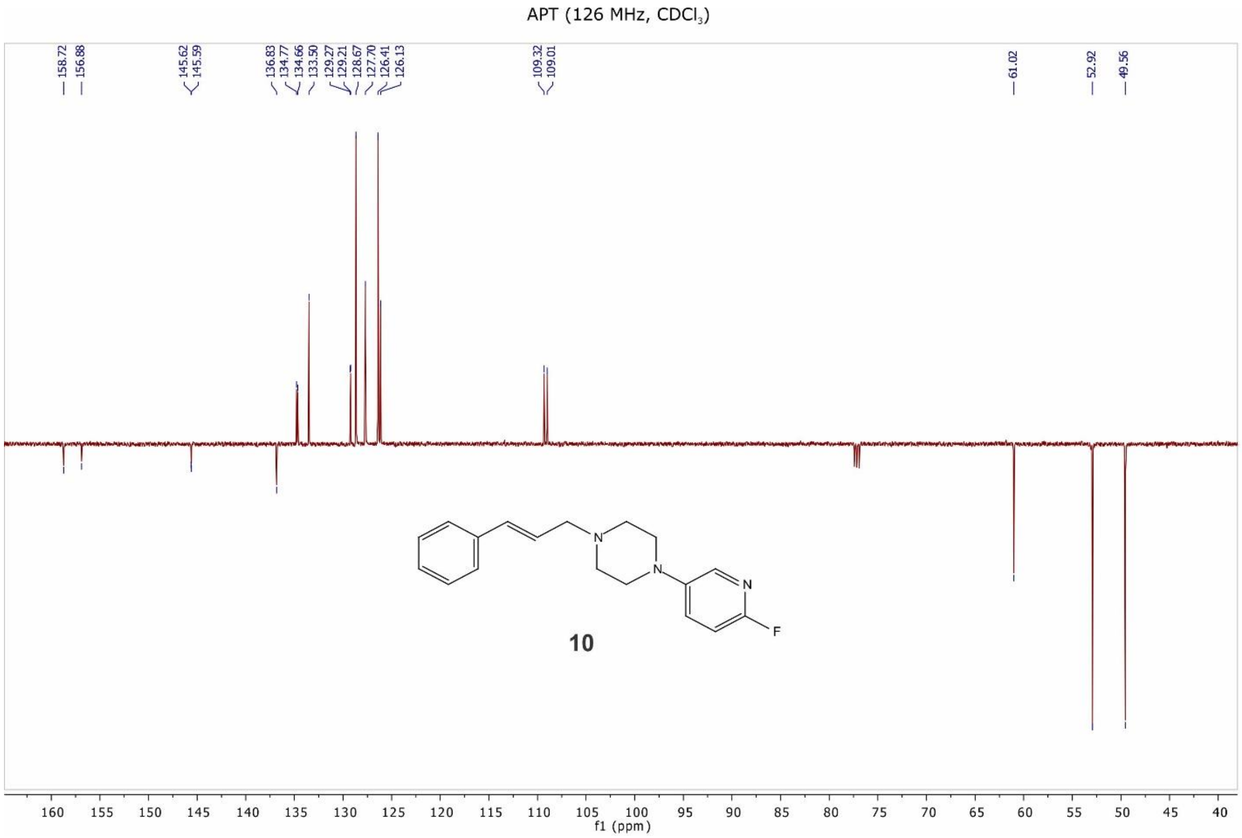
APT (126 MHz, CDCl3)
158.72
156.88
145.62
145.59
136.83
134.77
134.66
133.50
129.27
129.21
128.67
127.70
126.41
126.13
109.32
109.01
61.02
52.92
49.56
N
N
N
F
10
f1 (ppm)

HSQC ($CDCl_3$)

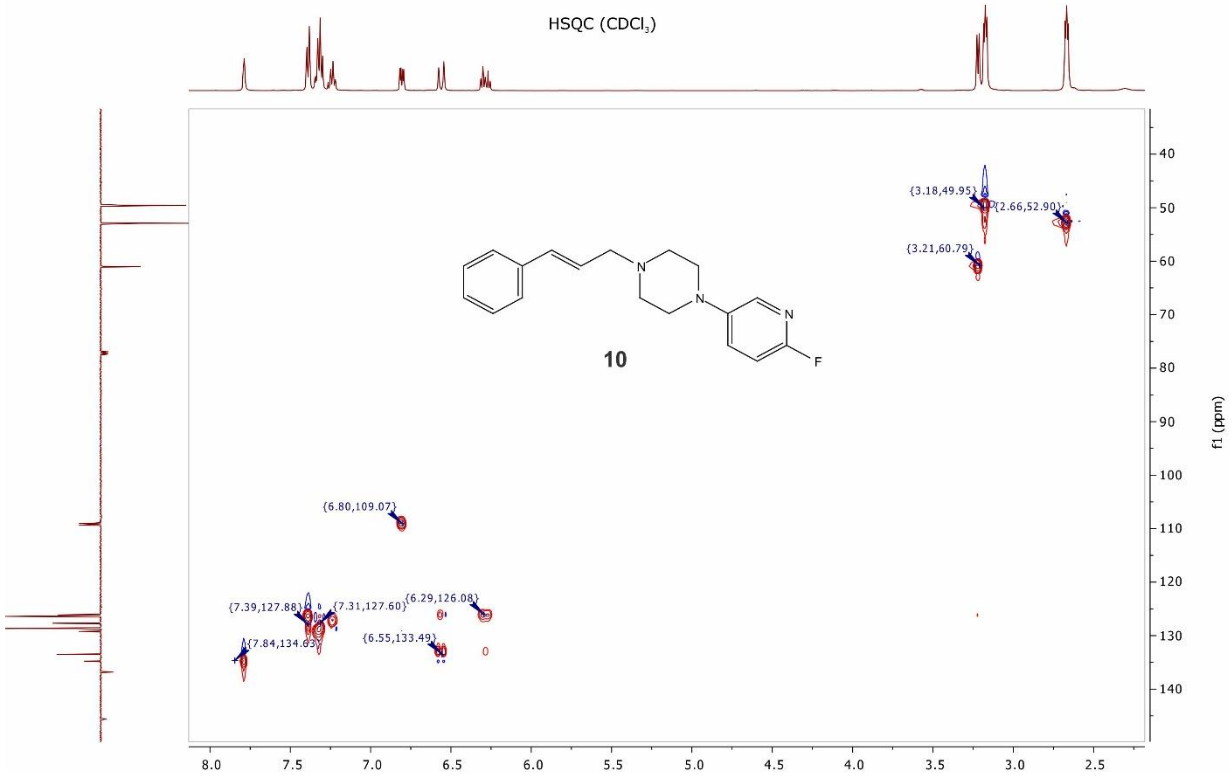

^1H NMR (500 MHz, $CDCl_3$)

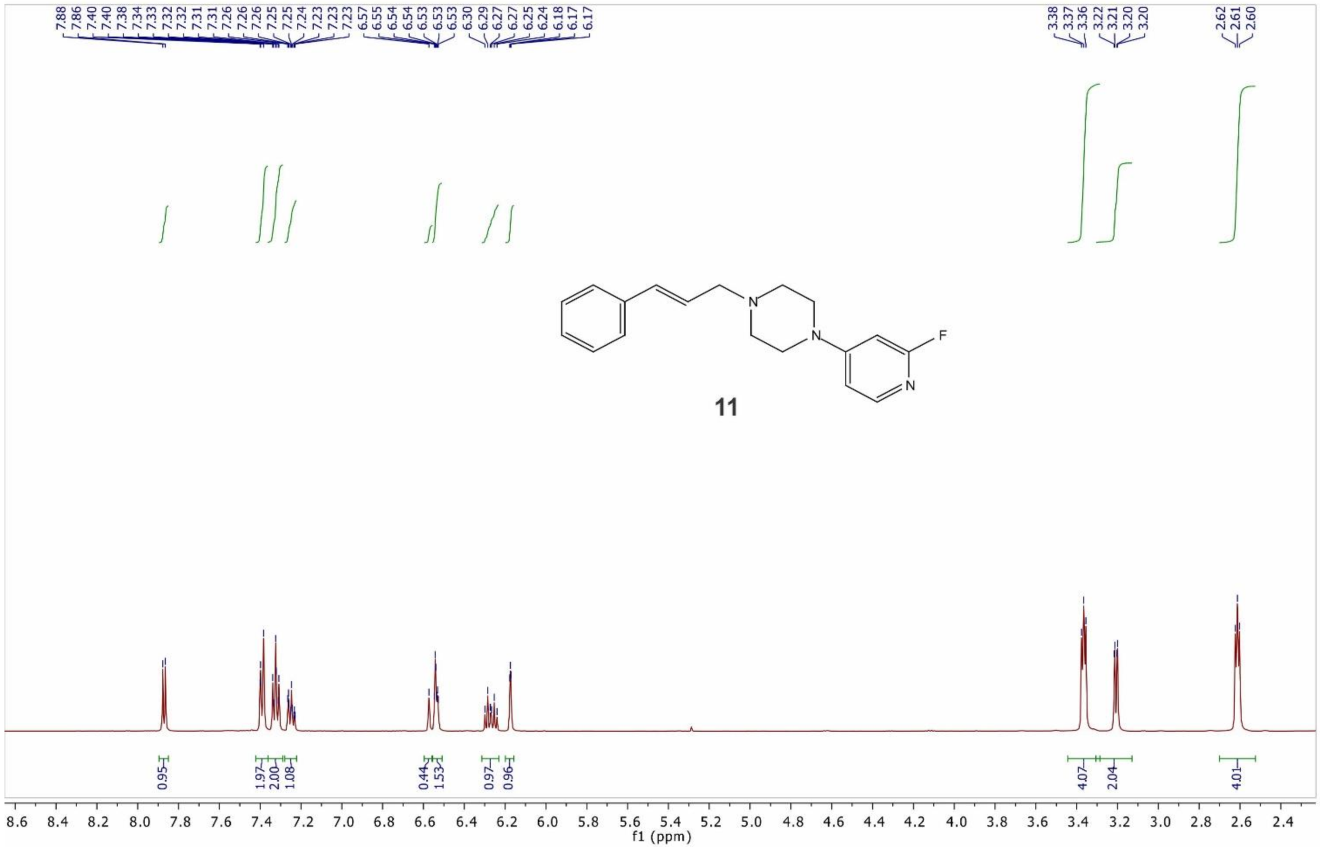

^{13}C NMR (126 MHz, $CDCl_3$)

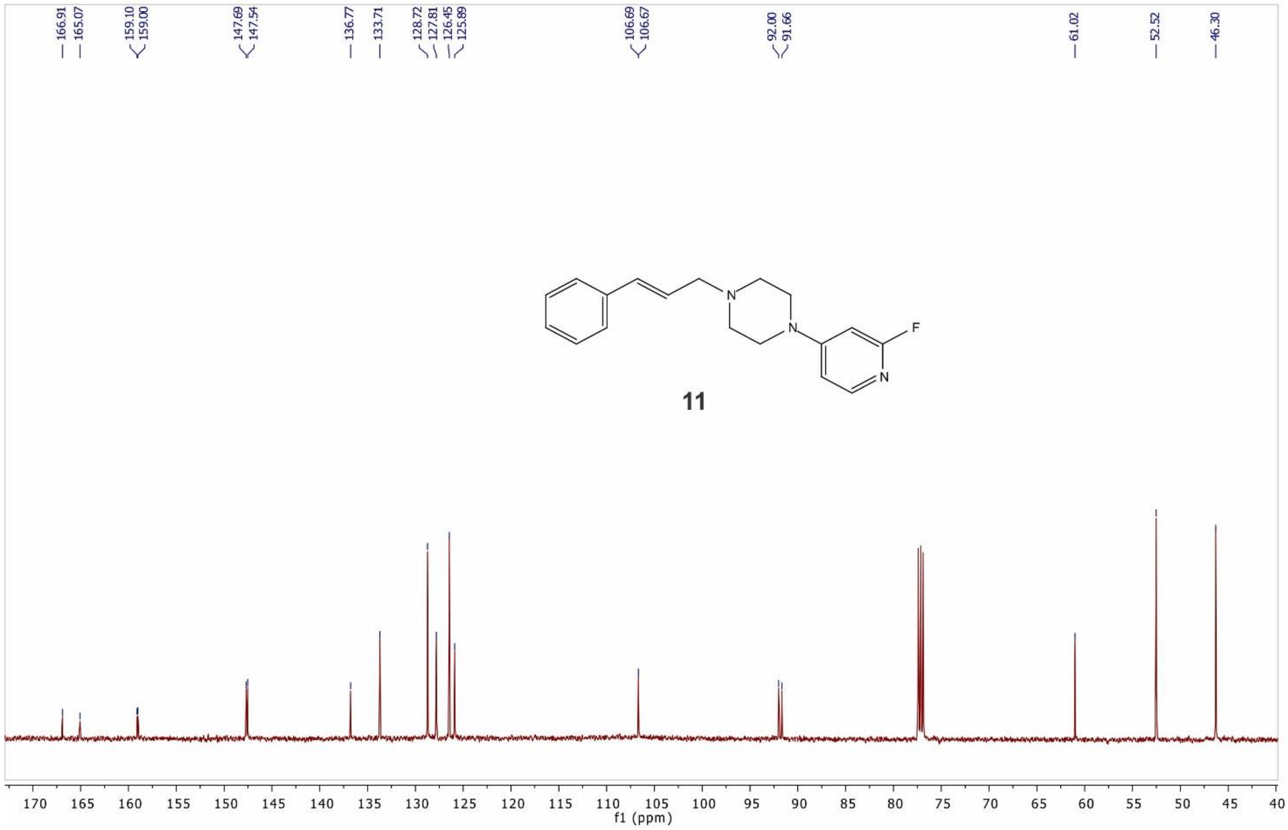

APT (126 MHz, $CDCl_3$)

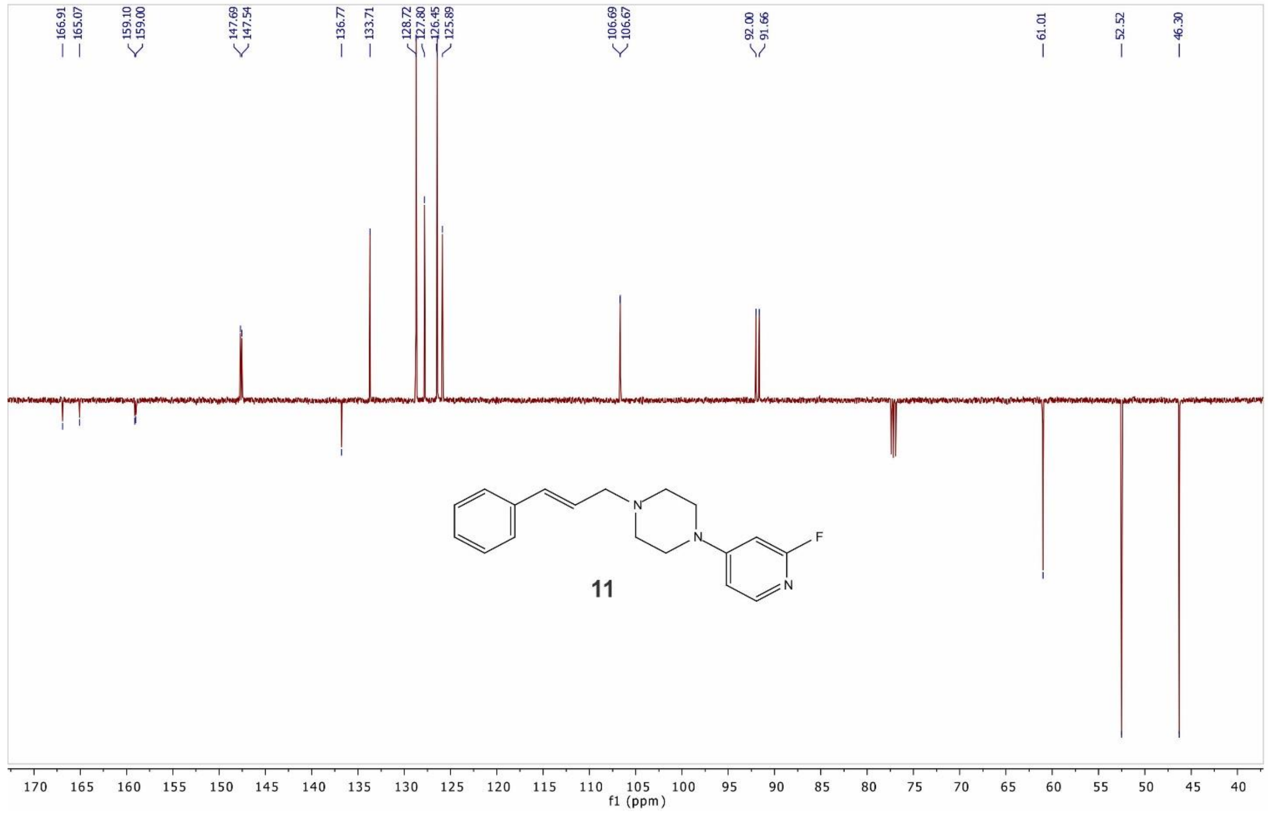

HSQC ($CDCl_3$)

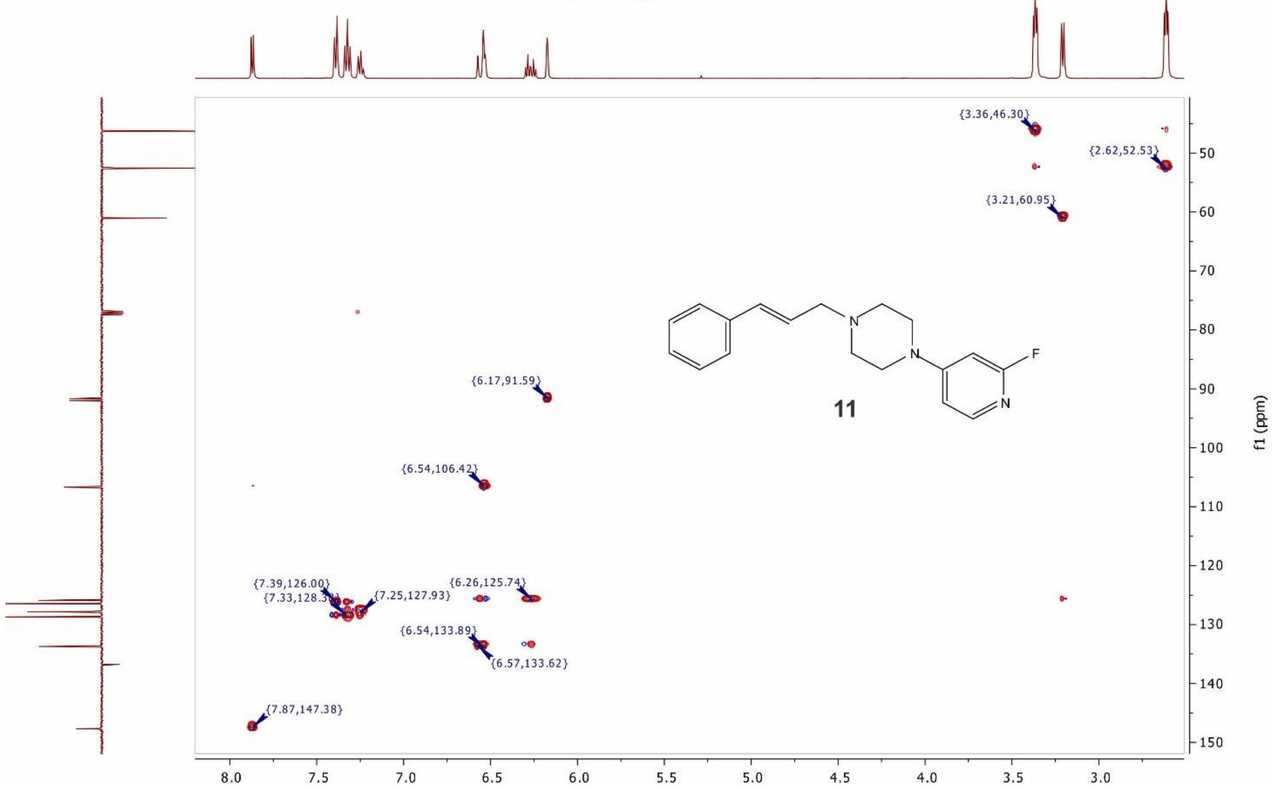

^{1}H NMR (500 MHz, $CDCl_3$)

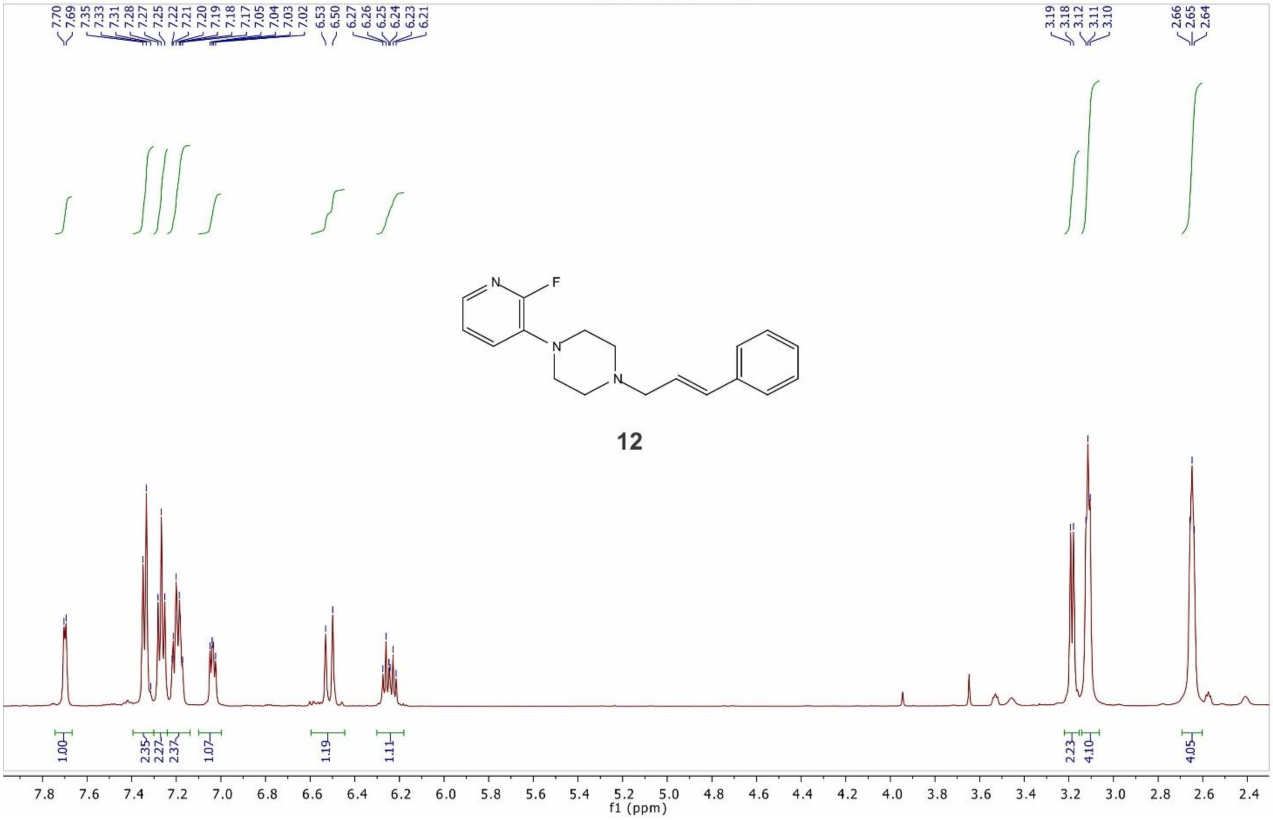

^{13}C NMR (126 MHz, $CDCl_3$)

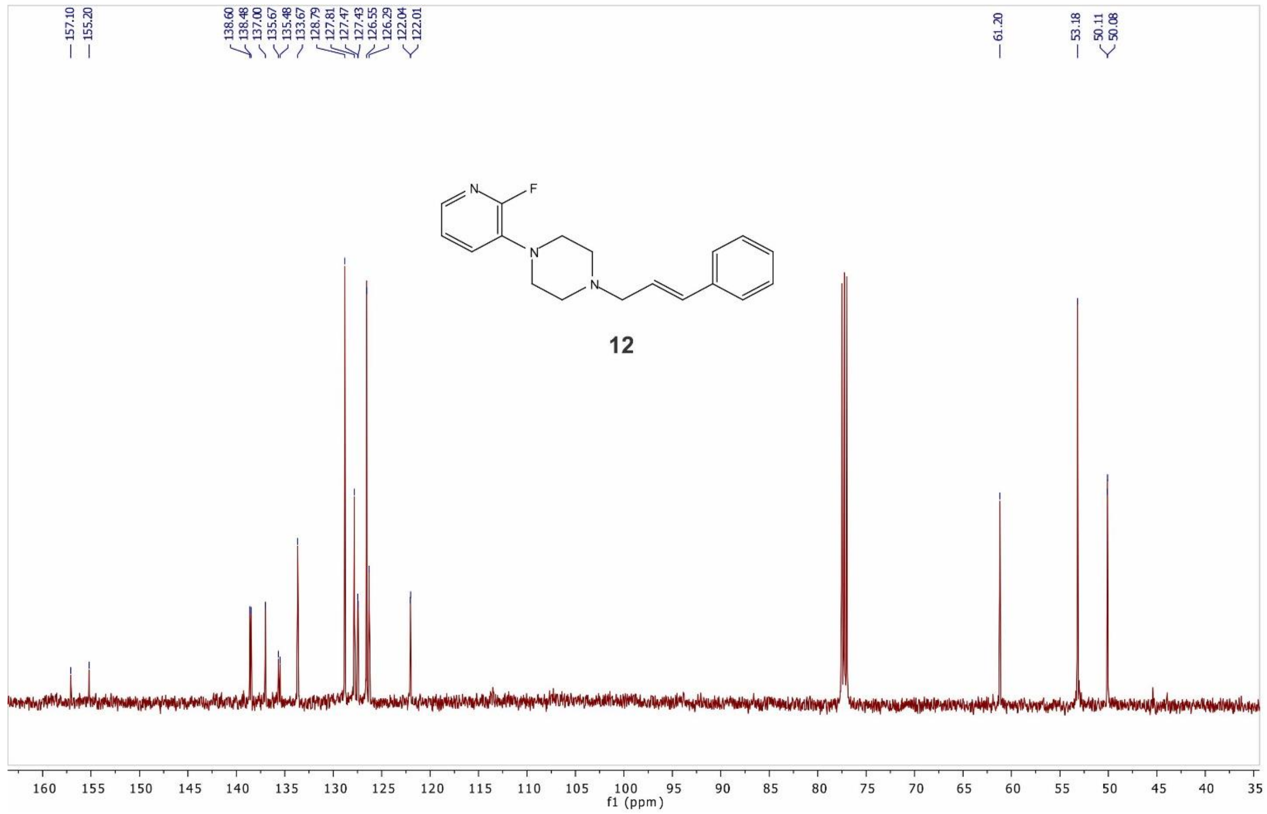

DEPT (126 MHz, $CDCl_3$)

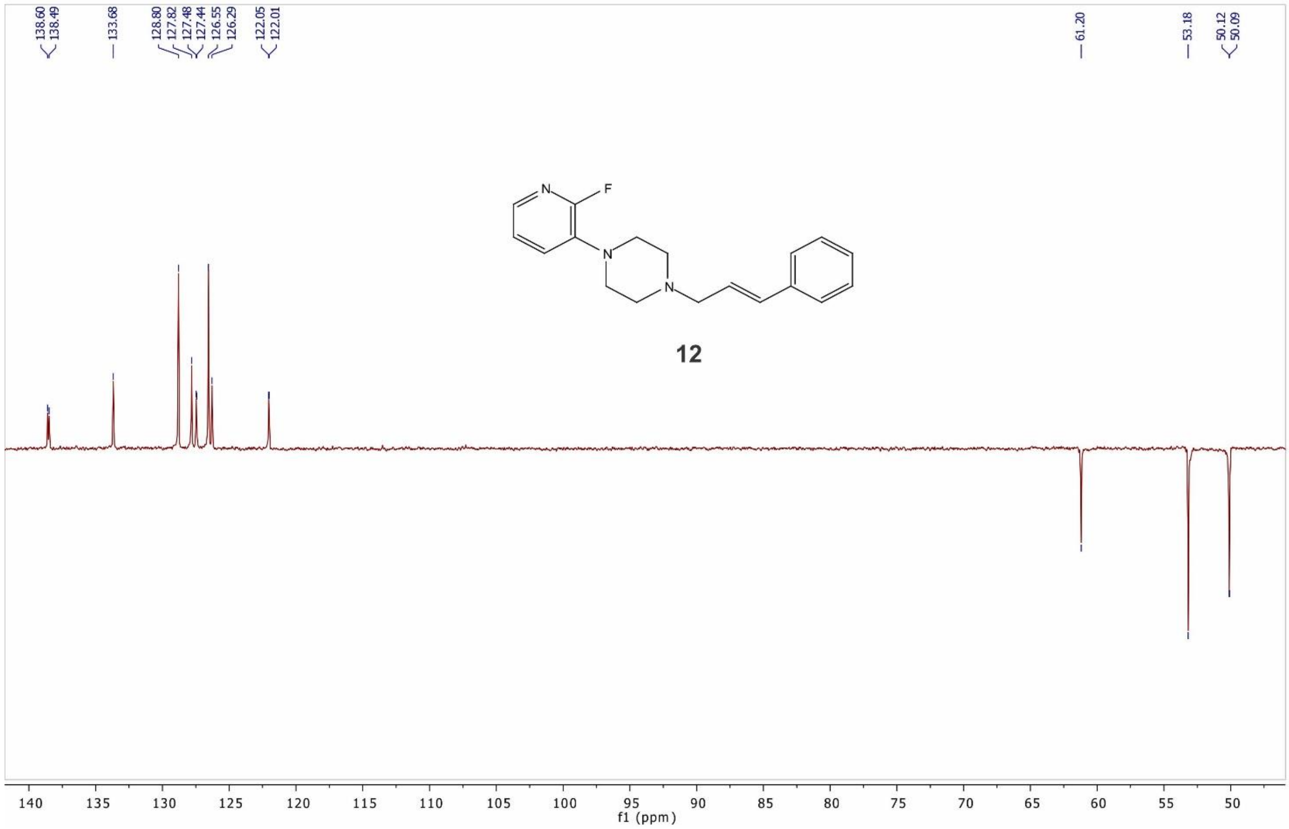

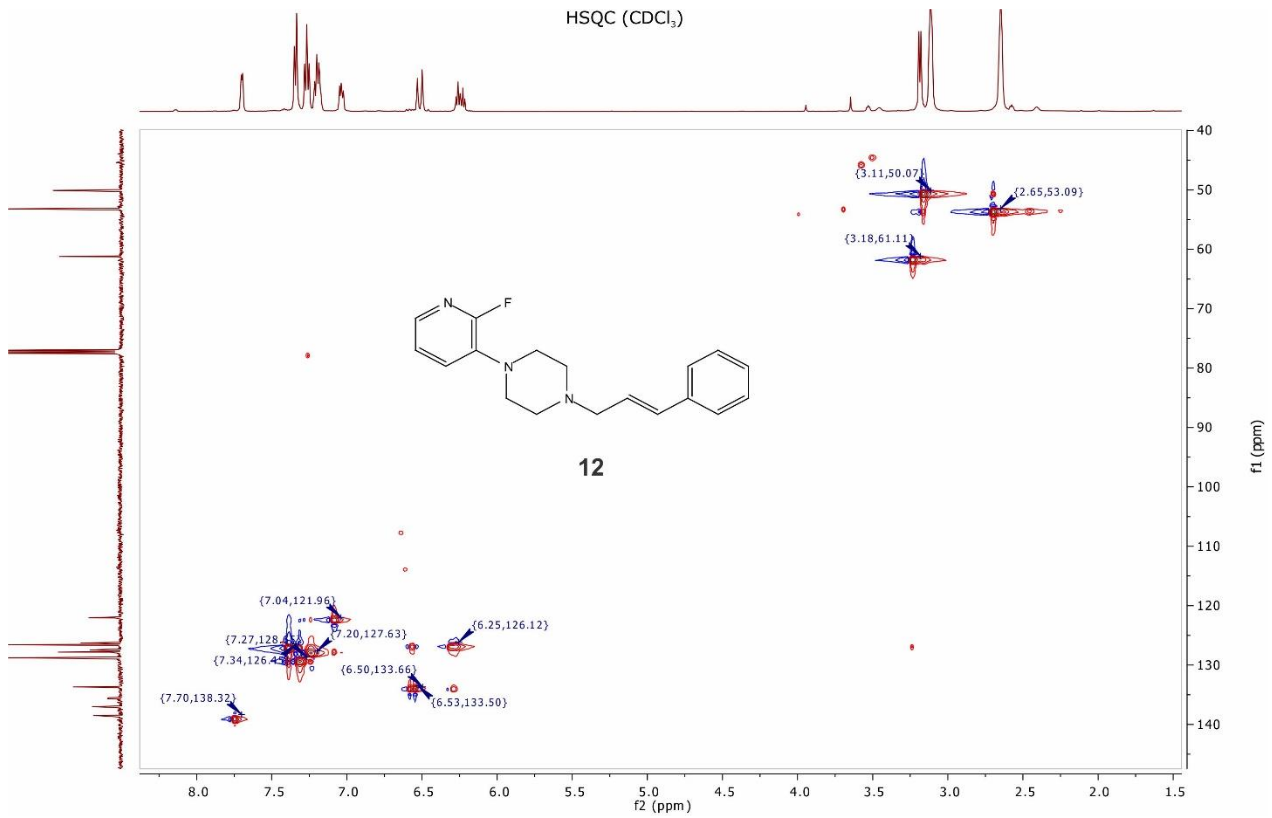
HSQC (CDCl$_3$)
12
f2 (ppm)
f1 (ppm)

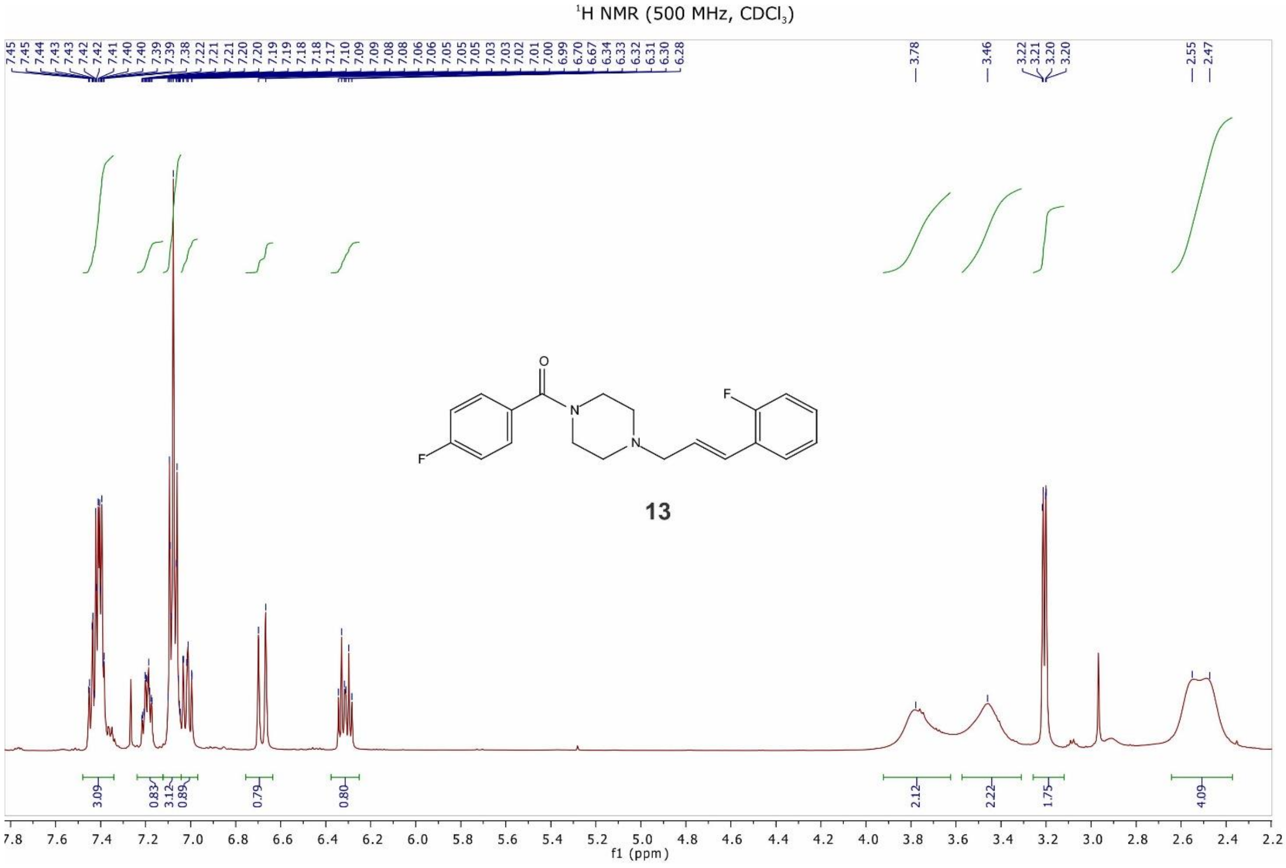
^{1}H NMR (500 MHz, CDCl$_3$)
13
f1 (ppm)

^{13}C NMR (126 MHz, $CDCl_3$)

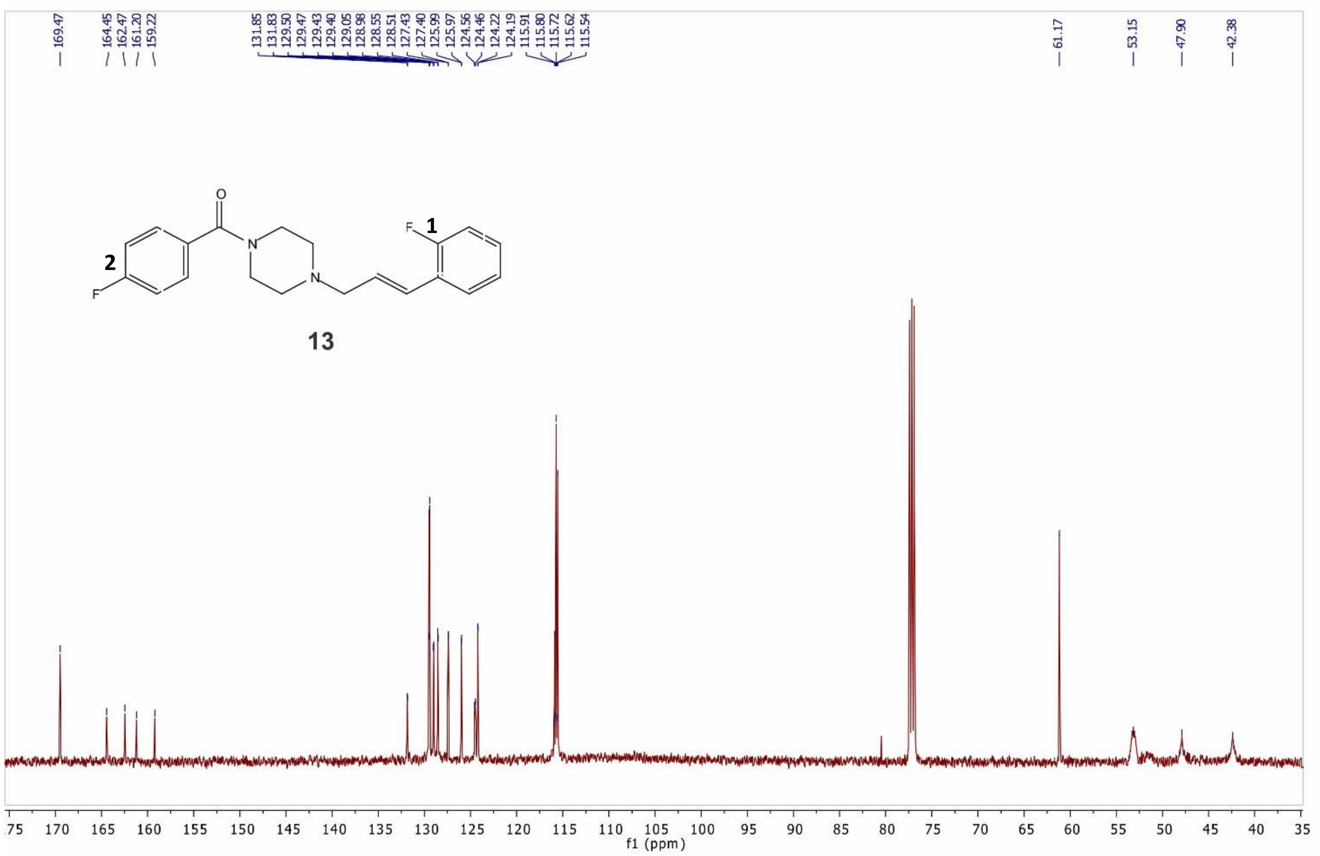

HSQC ($CDCl_3$)

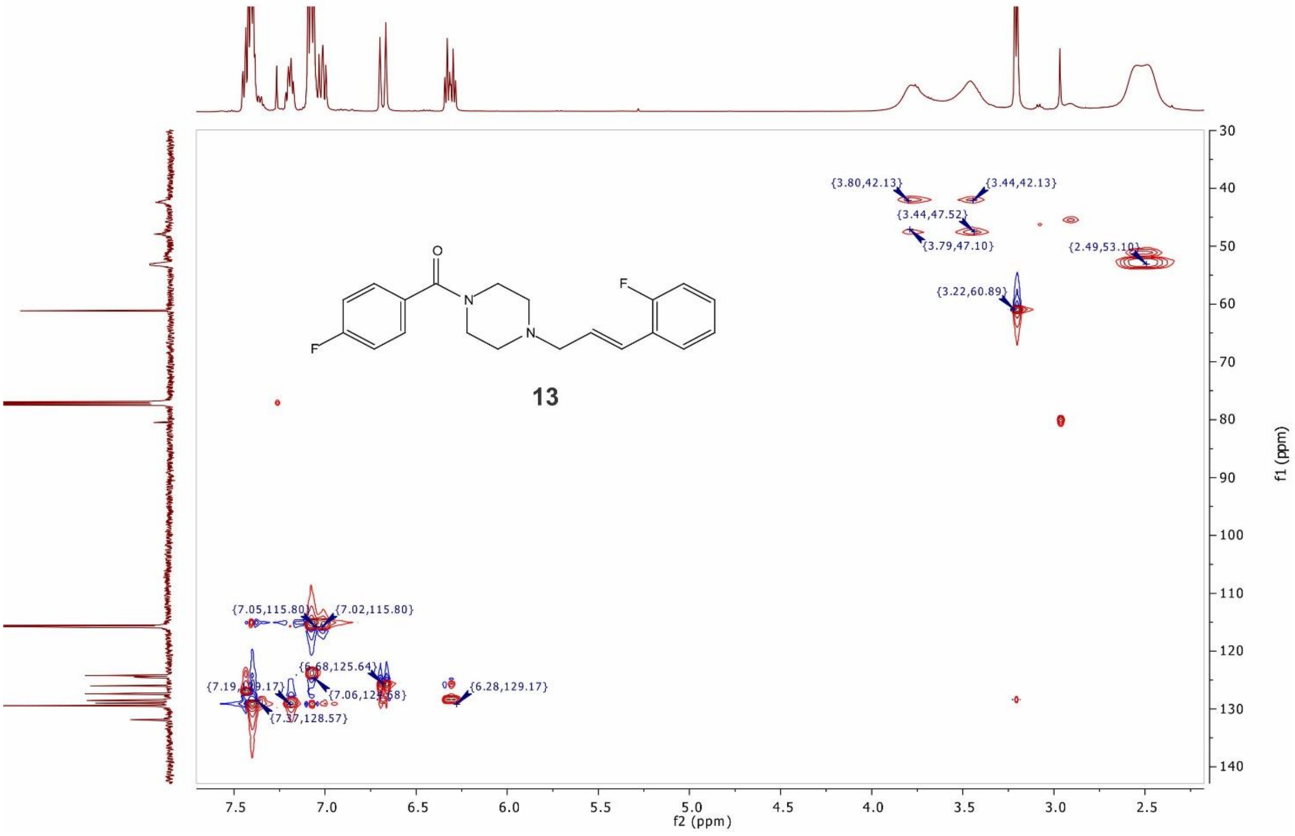

^{1}H NMR (500 MHz, $CDCl_3$)

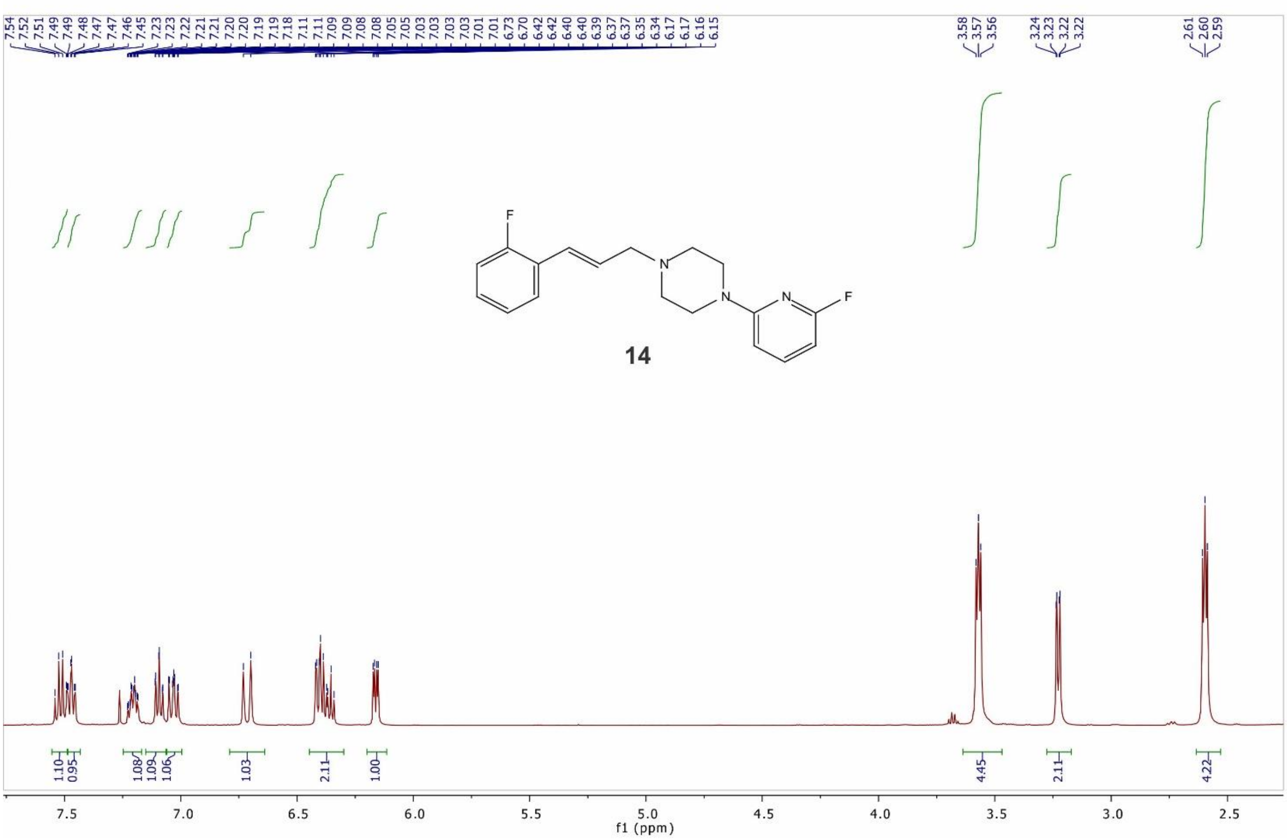

^{13}C NMR (126 MHz, $CDCl_3$)

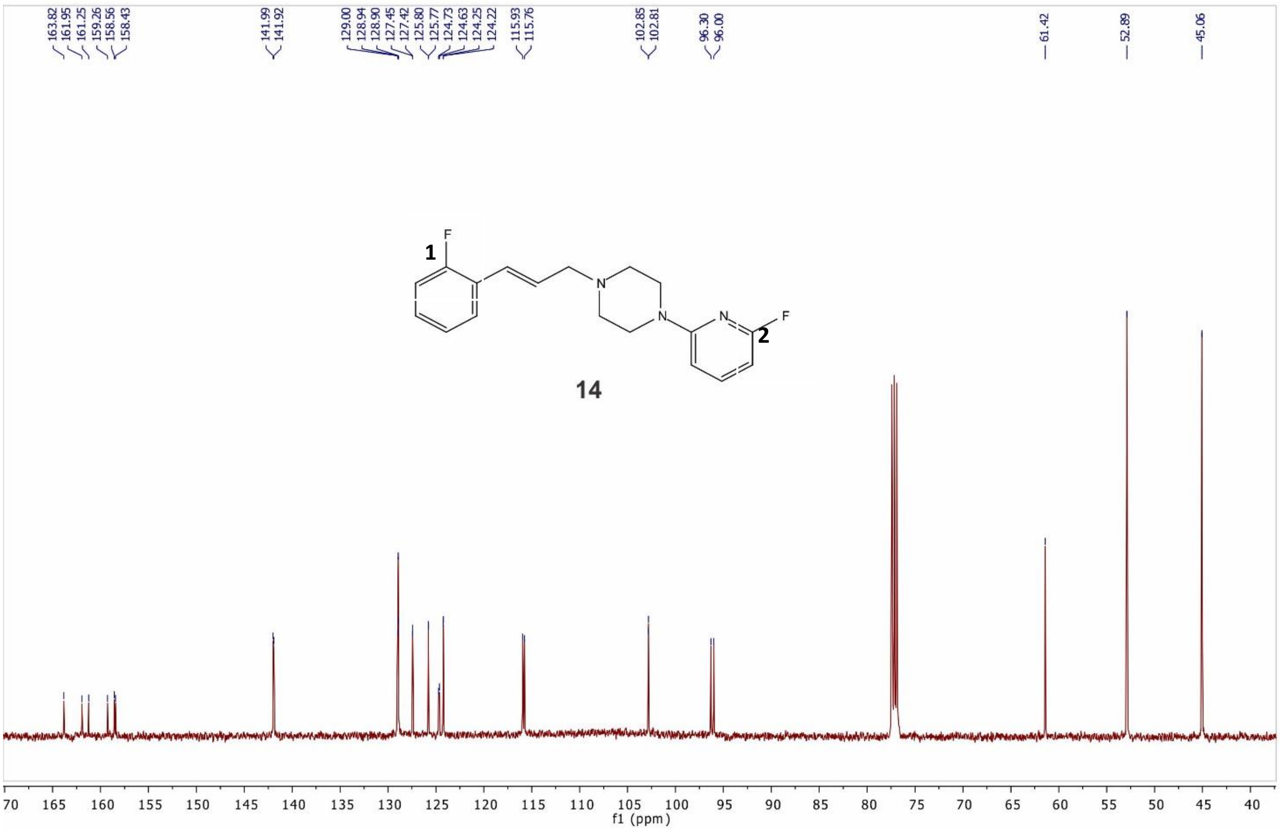

APT (126 MHz, $CDCl_3$)

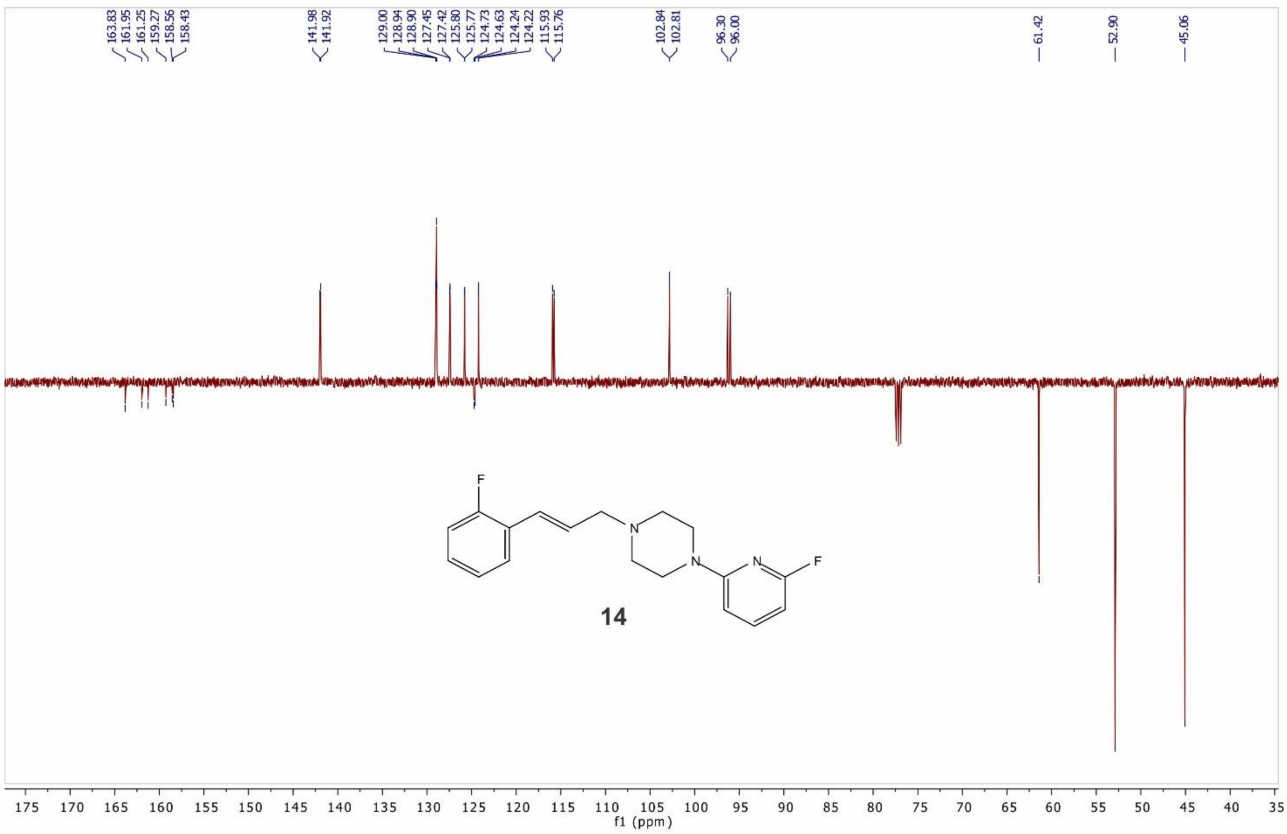

HSQC ($CDCl_3$)

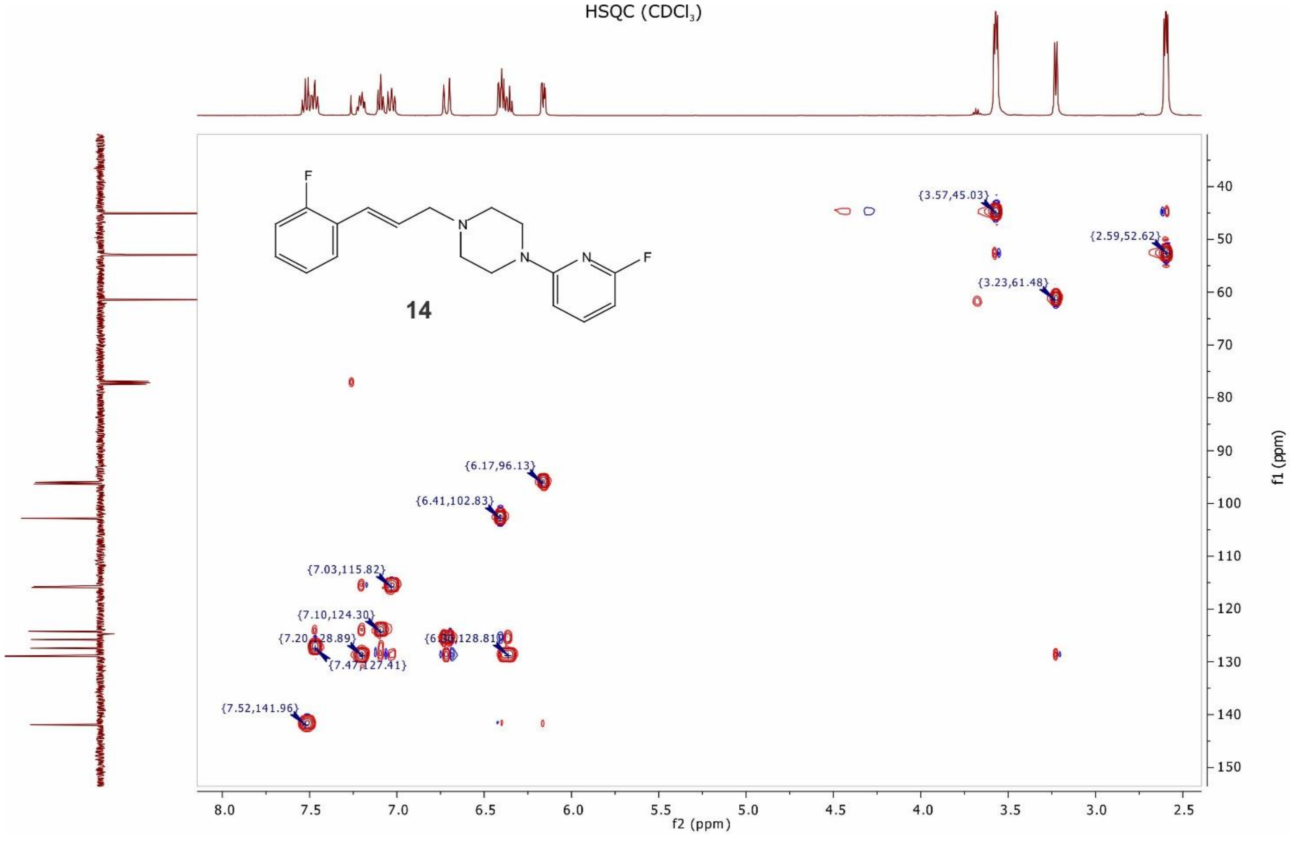

^{1}H NMR (500 MHz, $CDCl_3$)

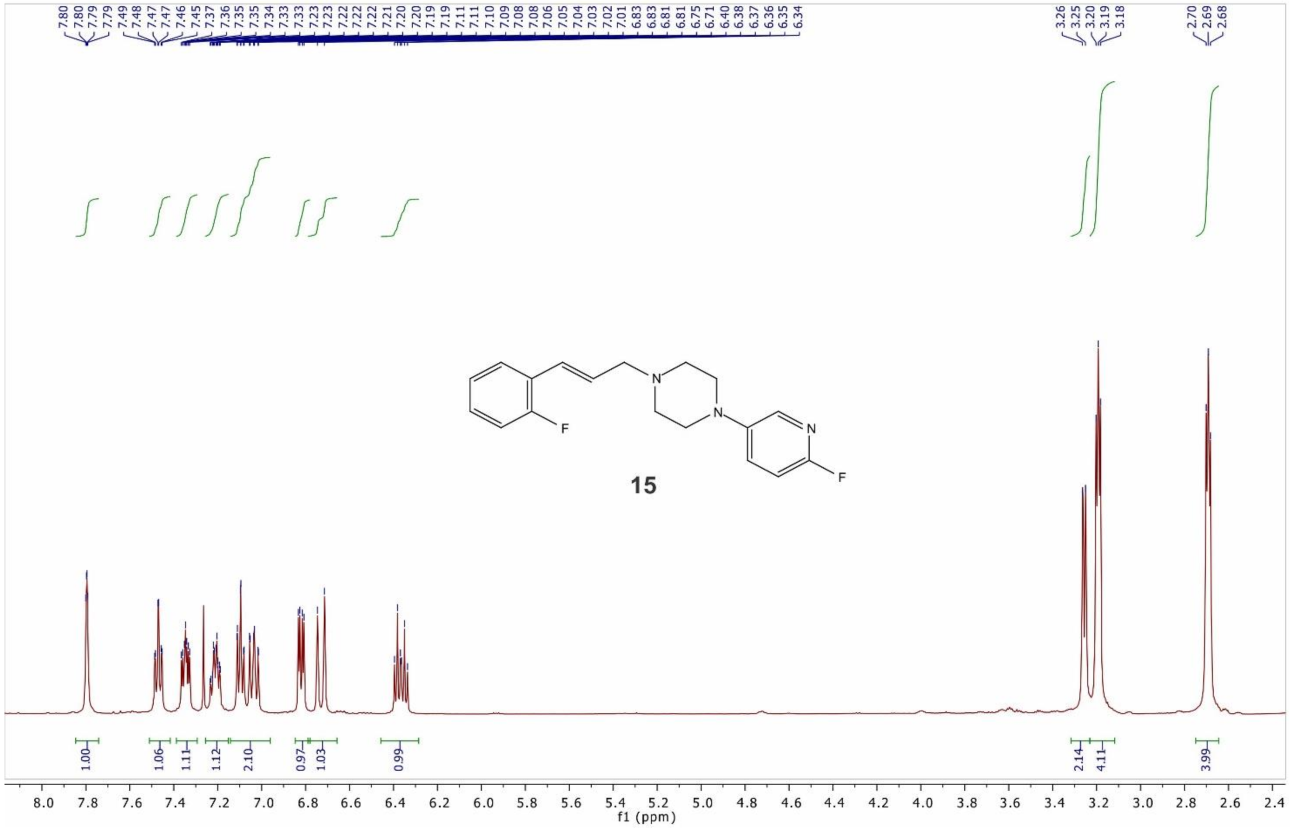

^{13}C NMR (126 MHz, $CDCl_3$)

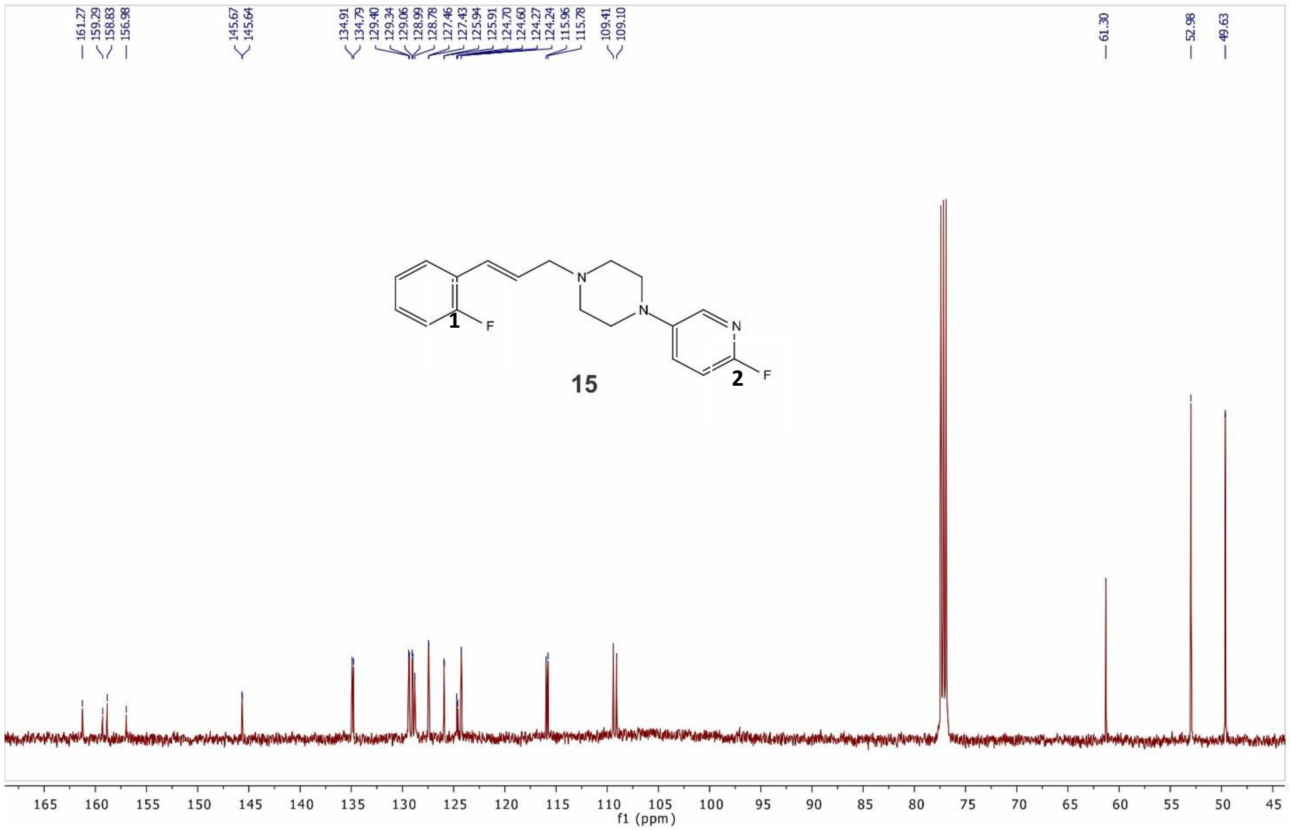

APT (126 MHz, $CDCl_3$)

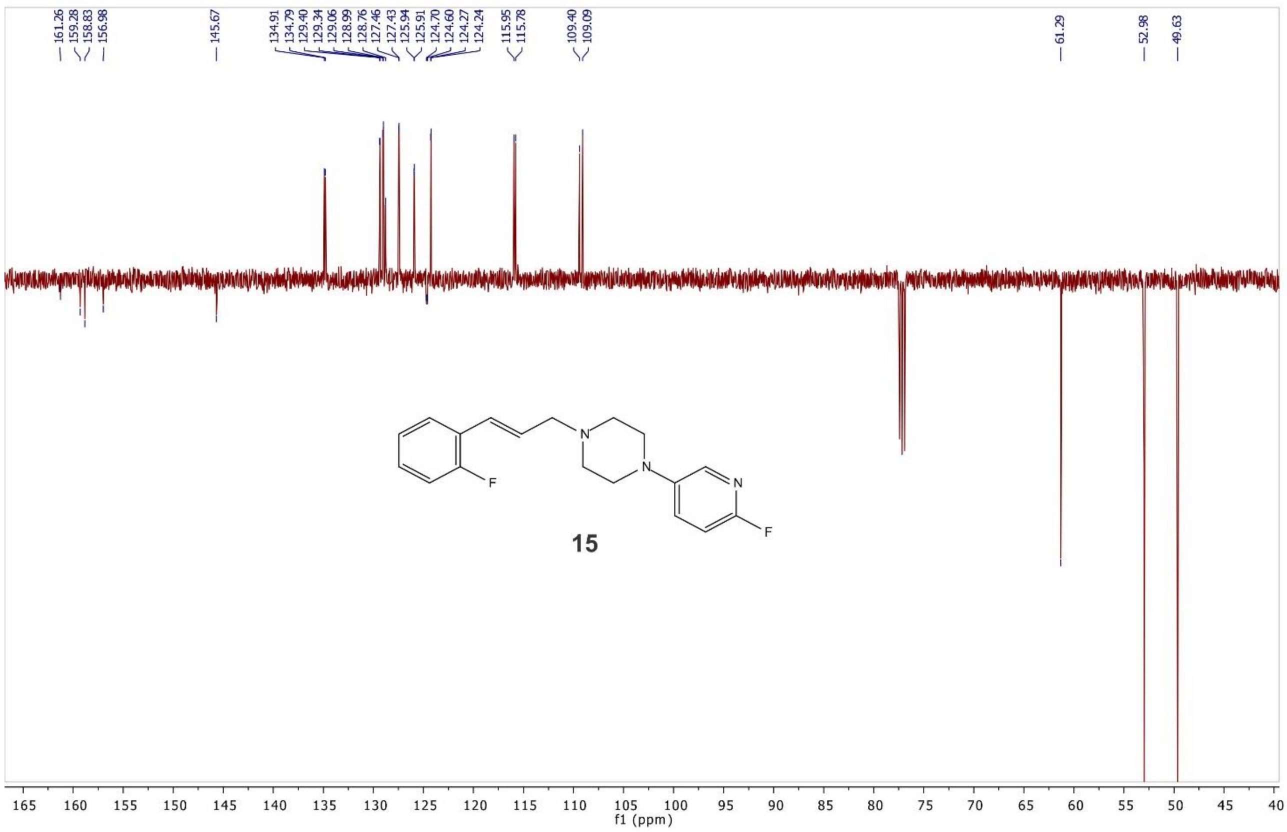

HSQC ($CDCl_3$)

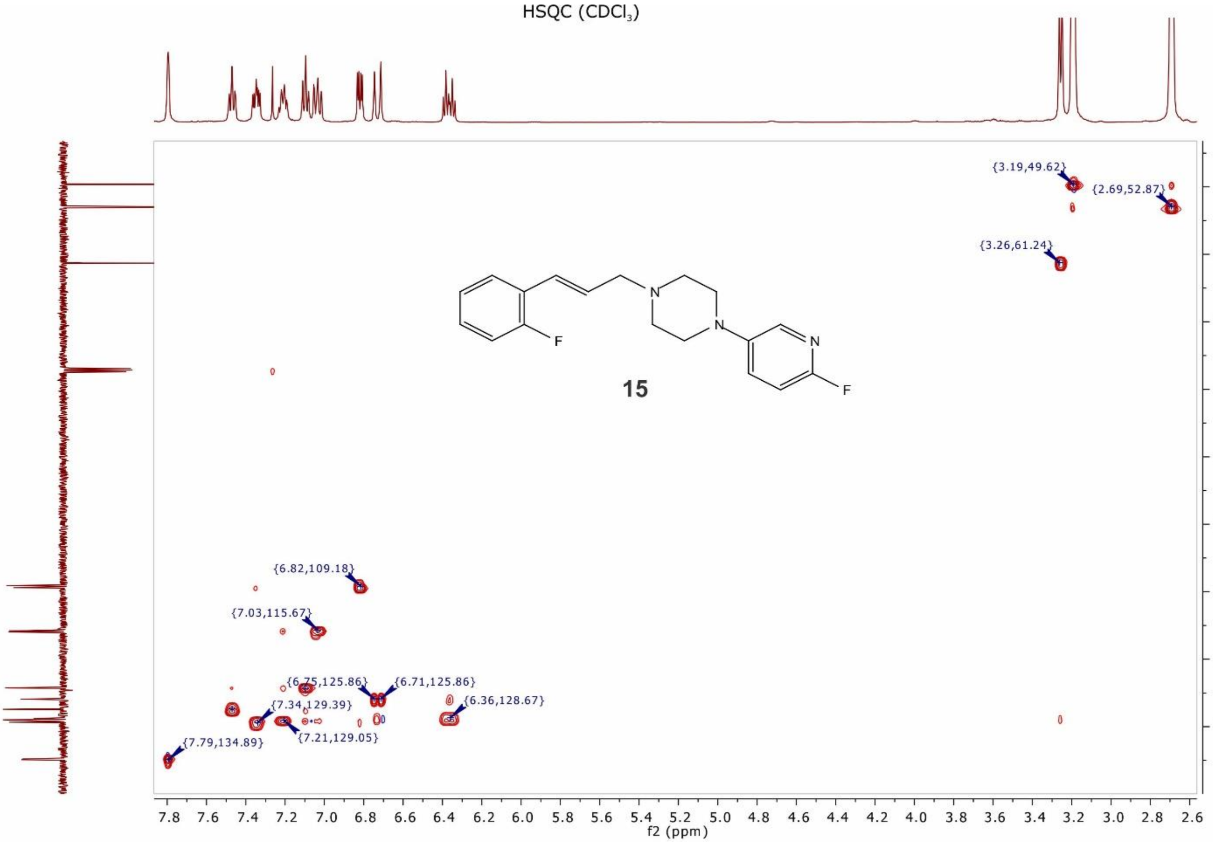

^{1}H NMR (500 MHz, $CDCl_3$)

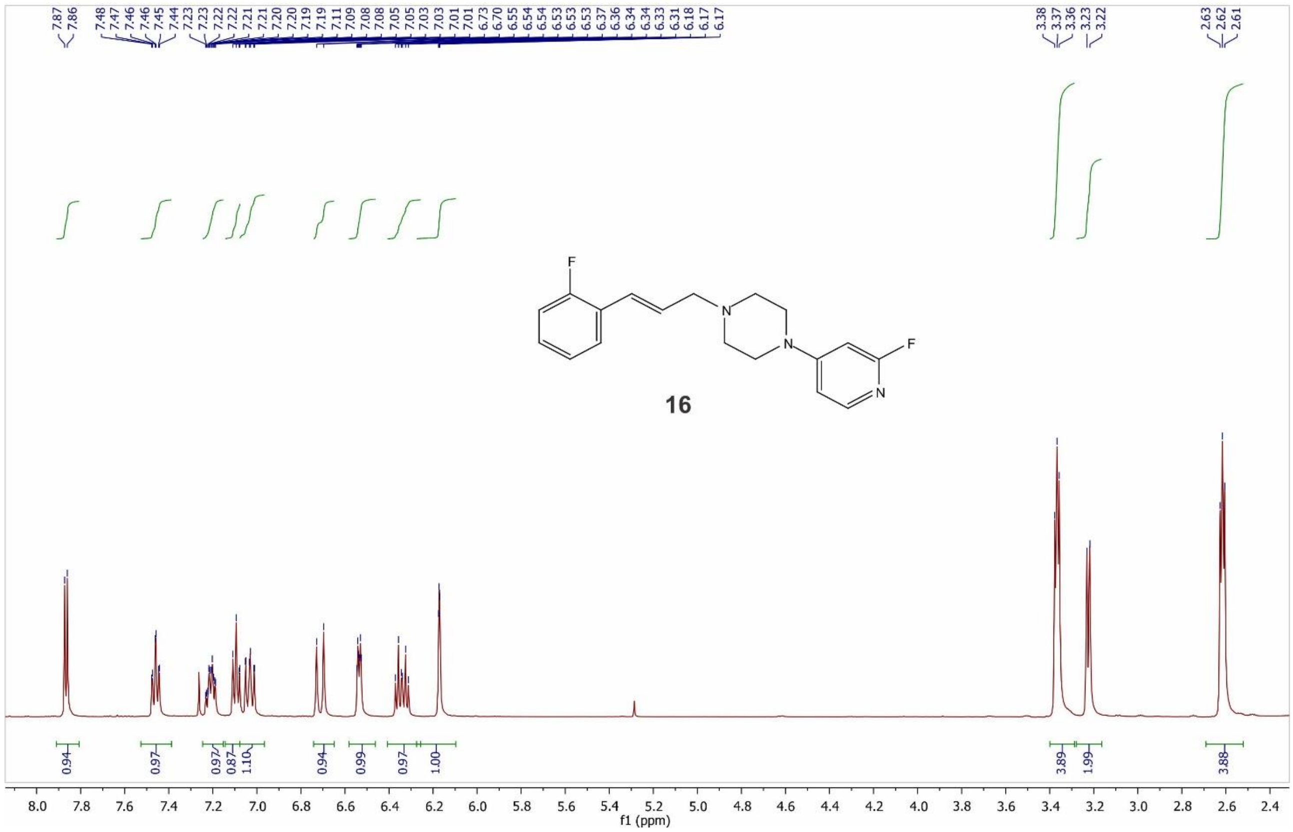

^{13}C NMR (126 MHz, $CDCl_3$)

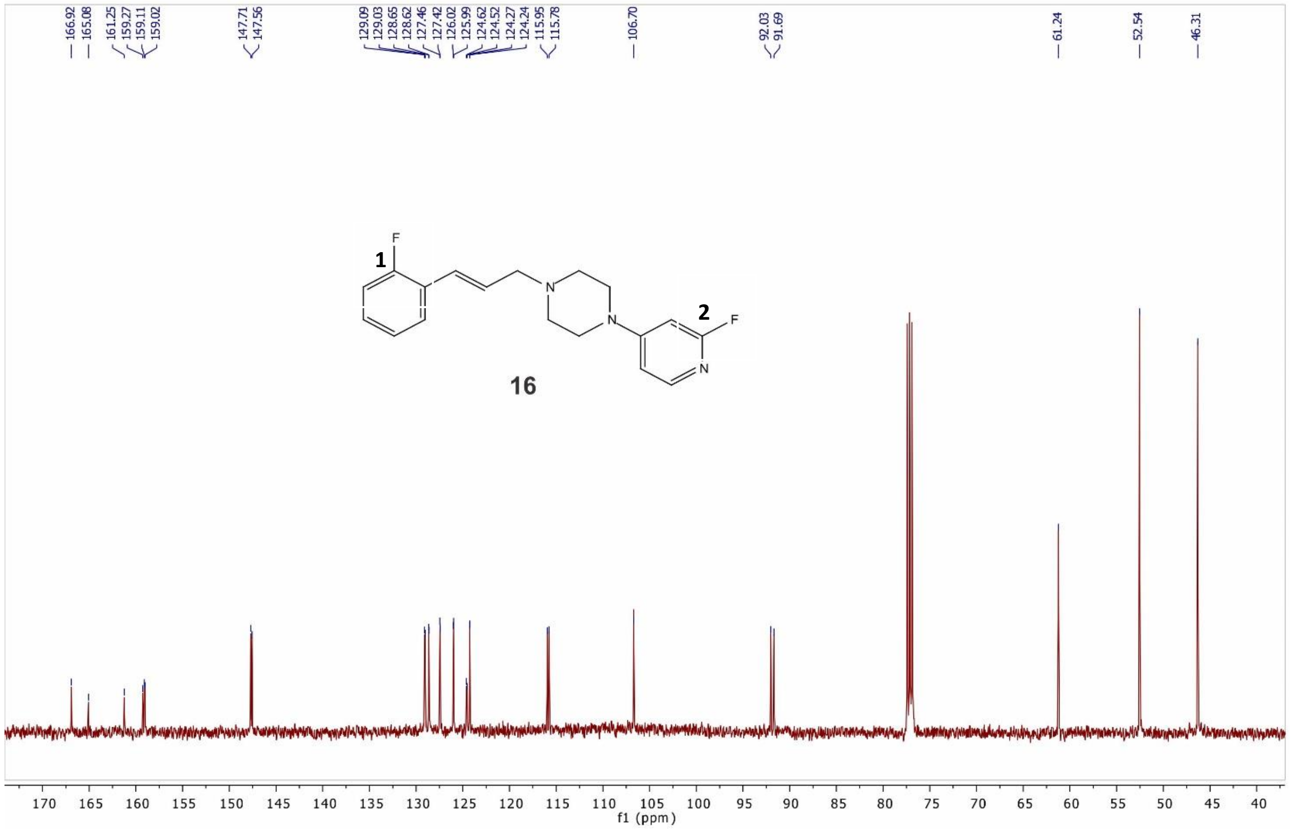

APT (126 MHz, $CDCl_3$)

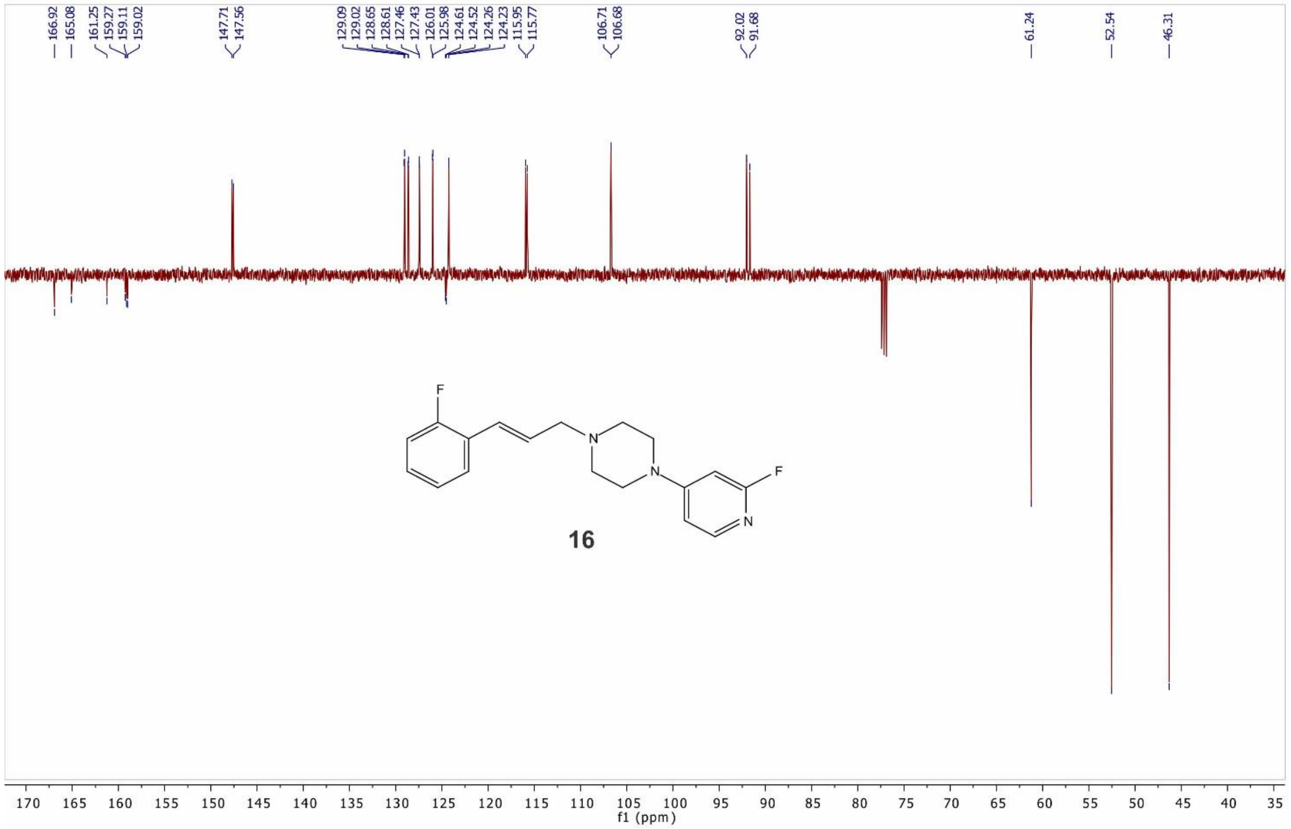

HSQC ($CDCl_3$)

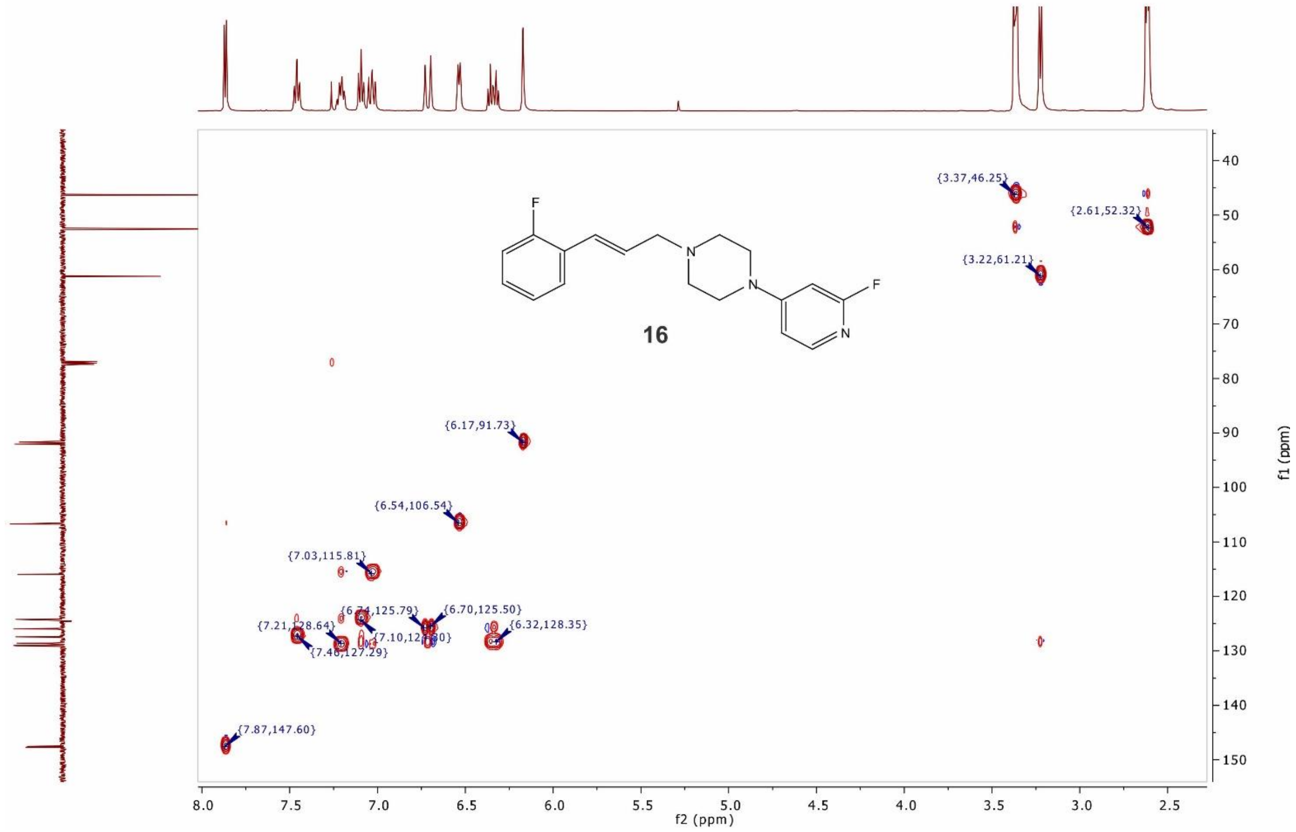

^{1}H NMR (500 MHz, $CDCl_3$)

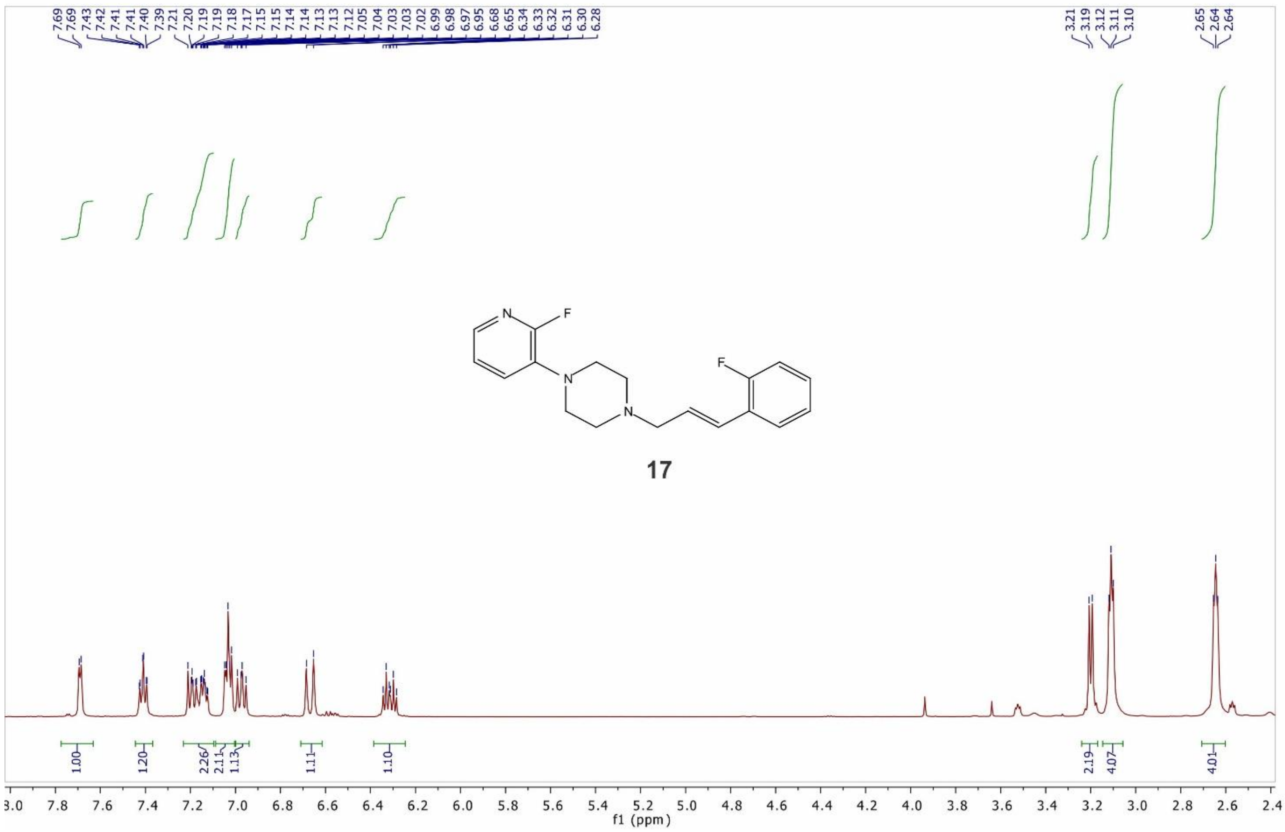

^{13}C NMR (126 MHz, $CDCl_3$)

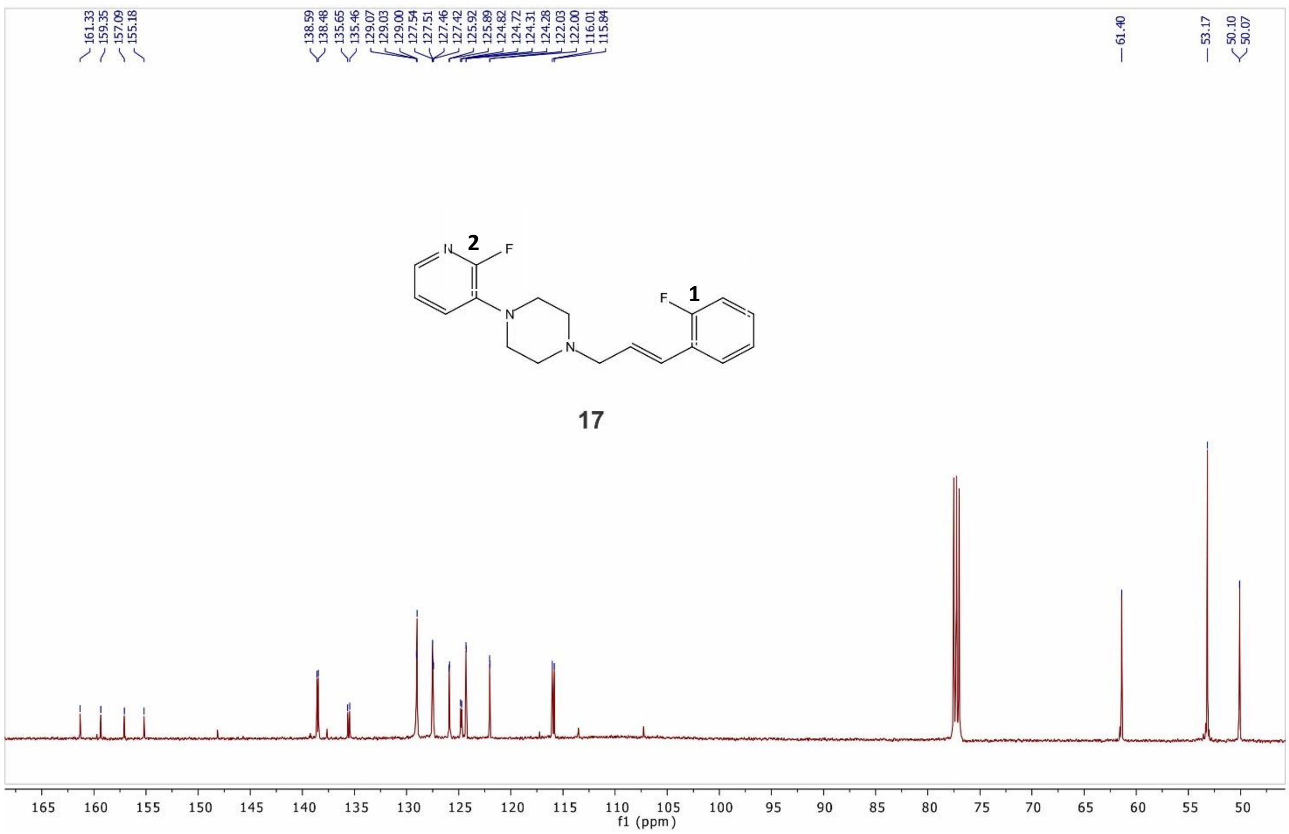

DEPT (126 MHz, $CDCl_3$)

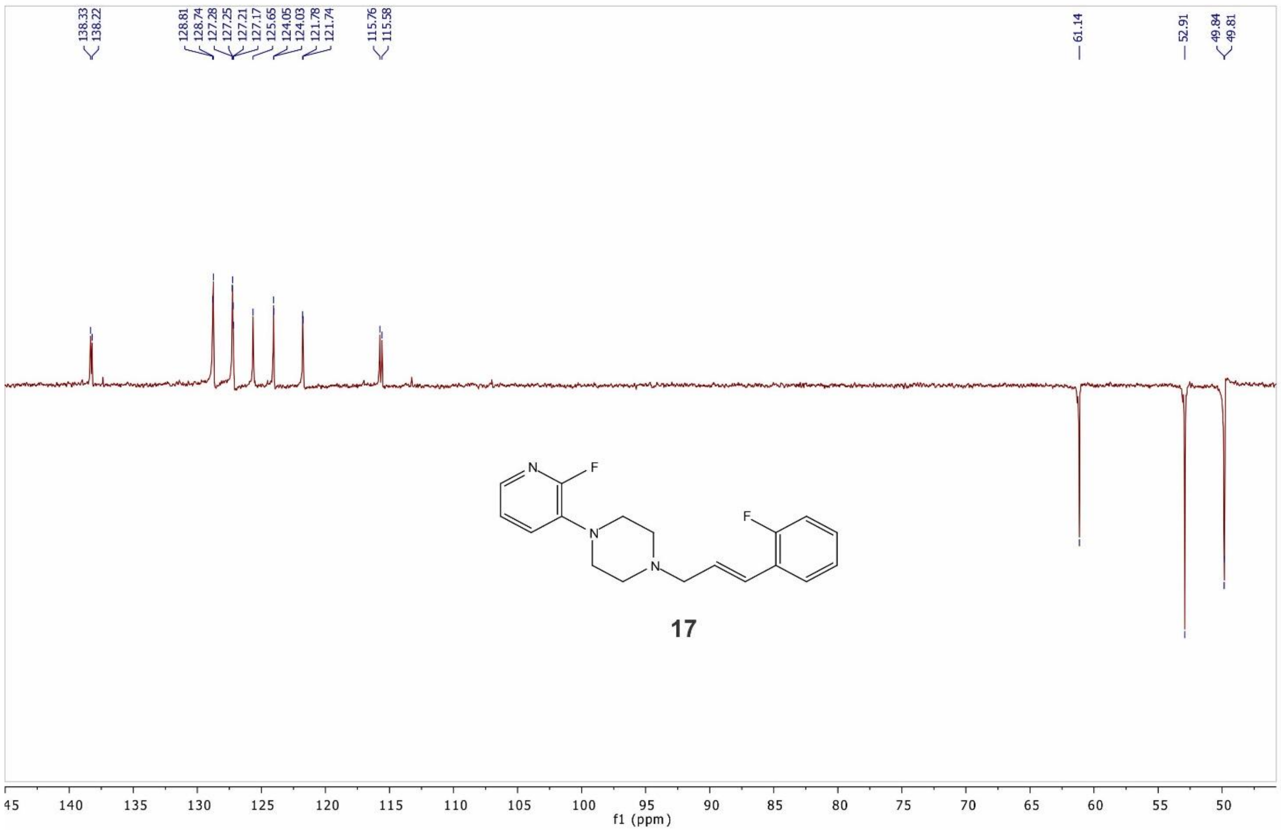

HSQC ($CDCl_3$)

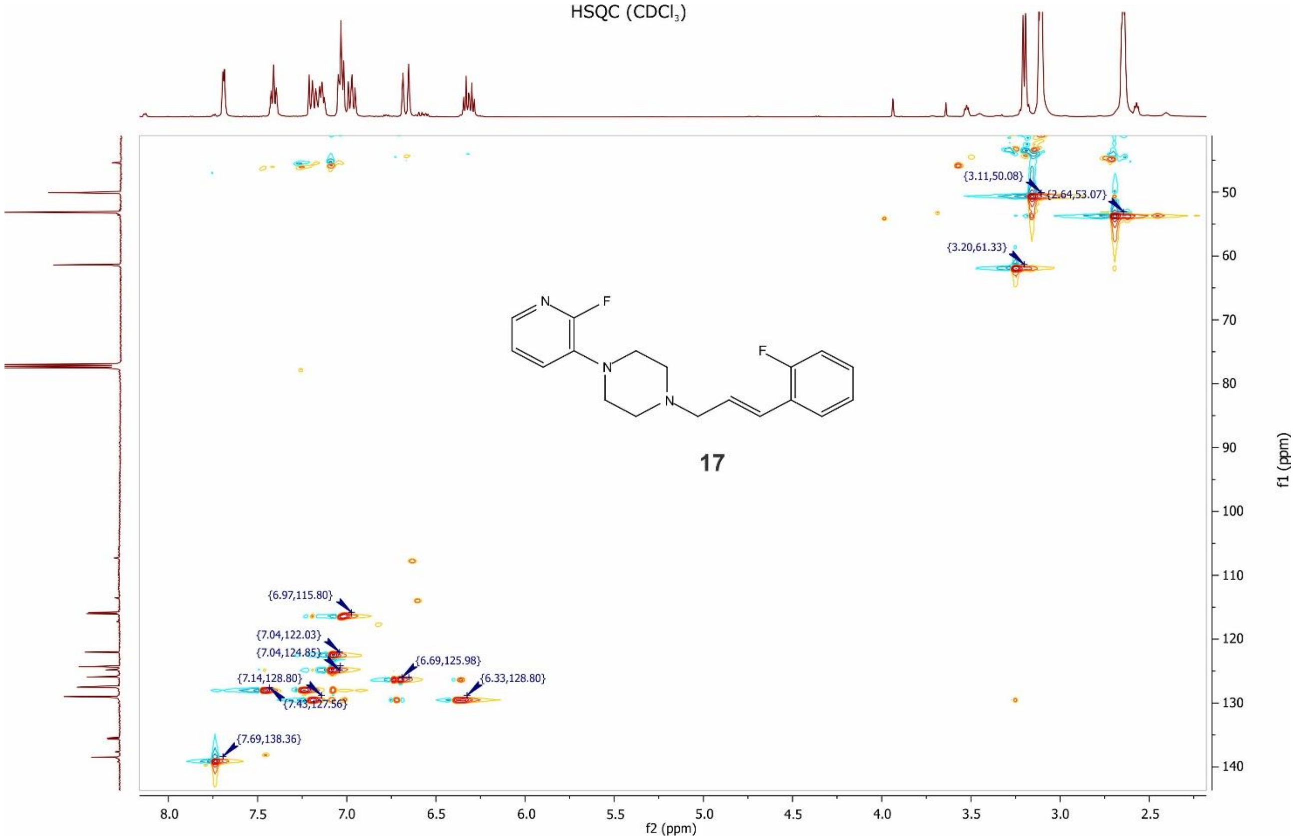

References:

1. A. K. Kumar, K. B. Nair, Y. D. Bodke, G. Sambasivam, G. Kishore, *Bhat Monatsh Chem*, **2016**, 147, 2221–2234.
2. J. Penjišević, V. Šukalović, D. Andrić, S. Kostić-Rajačić, V. Šoškić, G. Roglić, *Arch. Pharm. Chem. Life Sci.*, **2007**, 340, 456– 465.
3. B. Tae Cho, K. S. Kang, M. S. Kim, S. R. Ryub, D. Keun, *Tetrahedron*, **2006,** 62, 8164–8168.
4. L. Pimenta, E. V. Gusevskaya, E. E. Alberto, *Adv. Synth. Catal.*, **2017**, 359, 2297–2303.
